# A computational model of excitation and contraction in uterine myocytes from the pregnant rat

**DOI:** 10.1038/s41598-018-27069-x

**Published:** 2018-06-14

**Authors:** Craig P. Testrow, Arun V. Holden, Anatoly Shmygol, Henggui Zhang

**Affiliations:** 1 The University of Manchester, School of Physics and Astronomy, Manchester, M13 9PL UK; 2 The University of Leeds, School of Biomedical Sciences, Leeds, LS2 9JT UK; 3 United Arab Emirates University, College of Medicine and Health Sciences, Department of Physiology, Al-Ain, P.O. Box 17666 Emirates, UAE; 4 School of Computer Science and Technology, Harbin Institute of Technology (HIT), Harbin, 150001 China; 5 Space Institute of Southern China, Shenzhen, 518117 China; 6 Key laboratory of Medical Electrophysiology, Ministry of Education, Collaborative Innovation Center for Prevention and Treatment of Cardiovascular Disease/Institute of Cardiovascular Research, Southwest Medical University, Luzhou, 646000 China

## Abstract

Aberrant uterine myometrial activities in humans are major health issues. However, the cellular and tissue mechanism(s) that maintain the uterine myometrium at rest during gestation, and that initiate and maintain long-lasting uterine contractions during delivery are incompletely understood. In this study we construct a computational model for describing the electrical activity (simple and complex action potentials), intracellular calcium dynamics and mechanical contractions of isolated uterine myocytes from the pregnant rat. The model reproduces variant types of action potentials – from spikes with a smooth plateau, to spikes with an oscillatory plateau, to bursts of spikes – that are seen during late gestation under different physiological conditions. The effects of the hormones oestradiol (via reductions in calcium and potassium selective channel conductance), oxytocin (via an increase in intracellular calcium release) and the tocolytic nifedipine (via a block of L-type calcium channels currents) on action potentials and contractions are also reproduced, which quantitatively match to experimental data. All of these results validated the cell model development. In conclusion, the developed model provides a computational platform for further investigations of the ionic mechanism underlying the genesis and control of electrical and mechanical activities in the rat uterine myocytes.

## Introduction

Disorders in uterine excitation or contractility can lead to a range of complications for the mother and child, including preterm birth, ineffective and long labour, and post-partum haemorrhage. Premature births are associated with an increased chance of morbidity and mortality for the child and are the leading cause of death in children under 5 worldwide^1^. If they survive beyond five years these children tend to be small, lightweight and possibly suffering from mild to severe disabilities^2^. To reduce the risk of preterm birth, a better understanding of the mechanism(s) underlying the initiation and control of electrical and mechanical activities of the uterus is needed.

The electrophysiology of the human myometrium is complex, both in terms of how the cell and tissue properties change during gestation, and how electrical activity and mechanical contractions are initiated and maintained. In the past several decades, extensive experimental studies^3–10^ have been conducted to investigate in detail the cellular and tissue electrophysiology of the uterus. It has been shown that uterine mechanical contractions result from the integrated electrophysiology, biochemistry and biomechanics of uterine smooth muscle cells within the myometrial tissue, and their synchronization, with the mechanically passive supporting tissue and its architecture. Pace-making sites, which may play an important role in initiating uterine electrical activity triggering mechanical contraction, have been identified and mapped in the pregnant rat uterus^3^. However, the exact mechanism(s) underlying the initiation and control of uterine electrical and mechanical activities remains incompletely understood.

Biophysically detailed computational models of uterine cells provide an alternative approach to experimental studies to investigate possible mechanism(s) underlying the genesis of uterine electrical and mechanical activities. Over the past decade, several cell models for uterine smooth muscle cell electrophysiology have been developed. These include the historical Bursztyn *et al*.^11^ and Rihana *et al*.^12^ models that laid the foundation for current myometrial cell models. In 2011, Tong *et al*.^13^ developed the first comprehensive cell model for rat uterine cells that coupled cellular electrophysiology to the intracellular Ca^2+^ handling and the generation of active force of myofilament. The model was later updated to include some more potassium channels in 2014^14^. In 2016, Atia *et al*.^15^ developed the most comprehensive human myometrial electrophysiology model available, based on combining transcriptomic and biophysical data.

The aim of this study was to further develop and update a computational model for simulating the membrane potential and currents, intracellular calcium dynamics and mechanical activities of rat isolated uterine myocytes based on the Tong *et al*. 2011 model^13^ of rat uterine myocytes. First, the model was modified to incorporate some newly available experimental data on the kinetics of some membrane ion channels that underlie the membrane potential. This included reformulated equations for the L-type calcium current (I_CaL_), a voltage-activated potassium current (I_K2_), the calcium-activated potassium current (I_K(Ca)_) and the calcium-activated chloride current (I_Cl(Ca)_). Secondly, the model was modified to incorporate more detailed descriptions of the intracellular calcium handling and mechanical dynamics of the uterine cell, including a sarcoplasmic reticulum (SR). This enables the model to be related to cell and tissue recordings obtained by optical imaging of membrane potentials and intracellular calcium^16,17^. Finally, the developed model was validated by its ability to reproduce the functional impacts of hormone and drug actions, such as those of oestradiol, oxytocin and nifedipine on the electrical and mechanical behaviours of uterine cells. Table 1 summarises and compares the elements included in models previous to the one presented in this study (referred to as Testrow *et al*. 2018), highlighting the progress of cellular model development, especially in the areas of intracellular Ca^2+^ handling and cellular biomechanics.

**Table 1:** A summary of mammalian uterine smooth muscle cell models.

Model	Species	Channels					Pumps, exchangers	Sarcoplasmic reticulum	Ion handling	Mechanics
		Ca	Na	K	Cl	Other				
Bursztyn *et al*.^11^	Rat	CaL(V)	—	—	—	—	Ca, NaCa	—	Ca	7 ODEs
Rihana *et al*.^12^	Rat	CaL(V), CaT(V)	Na(V)	K(V), K(Ca), K-leak	—	—	—	—	—	—
Tong *et al*.^13^	Rat	CaL(V), CaT(V)	Na(V)	K1(V), K2(V), KA(V), K(Ca), K-leak	Cl(Ca)	h, NSCC(Ca, Na, K)	Ca, NaCa, NaK	—	Ca	1 ODE
Tong *et al*.^14^	Rat	CaL(V), CaT(V)	Na(V)	K1(V), K2(V), KA(V), K(Ca), KCNQ1, KCNQ4, KCNQ5, K(hERG), K-leak	Cl(Ca)	h, NSCC(Ca, Na, K)	Ca, NaCa, NaK	—	Ca	1 ODE
Atia *et al*.^15^	Human	CaL(V), CaT(V)	—	Kv2.1, Kv2.1 + Kv6.1, Kv2.1 + Kv9.3, Kv3.4, Kv4.1, Kv4.3 + KCNE3, Kv4.3 + KChIP2b/d, Kv4.3 + KChIP2b + KCNE3, Kv7.1, Kv7.4, Kir7.1, SK2–3, SK4, BK, BKβ1, BKβ3, BKβ4,K(hERG), bgK	Cl(Ca), bgCl	—	Ca, NaCa, NaK	—	Ca	—
Testrow *et* *al*. 2018	Rat	CaL(V), CaT(V)	Na(V)	K1(V), K2(V), KA(V), K(Ca), K-leak	Cl(Ca)	h, SOC(Ca, Na), NSCC(Ca, Na, K)	Ca, NaCa, NaK, NaKCl	SERCA, RYR	Ca, Na	7 ODEs

The channels are identified by their common names or their channel protein. These models all use quantitative descriptions of membrane ionic currents that have been derived from membrane and cell patch clamp data obtained under different conditions.

The paper is structured in the following way. In section 2, a brief description of the model development is presented (additional information can be found in the supplementary material). In section 3, simulation results are presented to demonstrate capability of the model in reproducing experimentally observed uterine cellular electrical and mechanical activities, including the spike train action potentials and staircase calcium transients associated with uterine cells. In addition, we demonstrated the model’s capability in reproducing the effects of some drugs and hormones on cellular electrical and mechanical activities. All of these simulation results qualitatively and quantitatively match to experimental data, validating the cell model development. With the validated cell model, we theoretically investigated the role of changing Na^+^ and Ca^2+^ ion channel current density in modulating cellular action potentials, the intracellular Ca^2+^ handling and the genesis of active force, exploring effects of possible ion channel remodelling during the gestational period on uterine cellular electrical and mechanical activities. In Section 4, we discuss the major contributions of this study.

## Model Development

This section presents a brief overview of the model development; full details of the model components are provided in the Supplementary Material.

**Figure 1:**
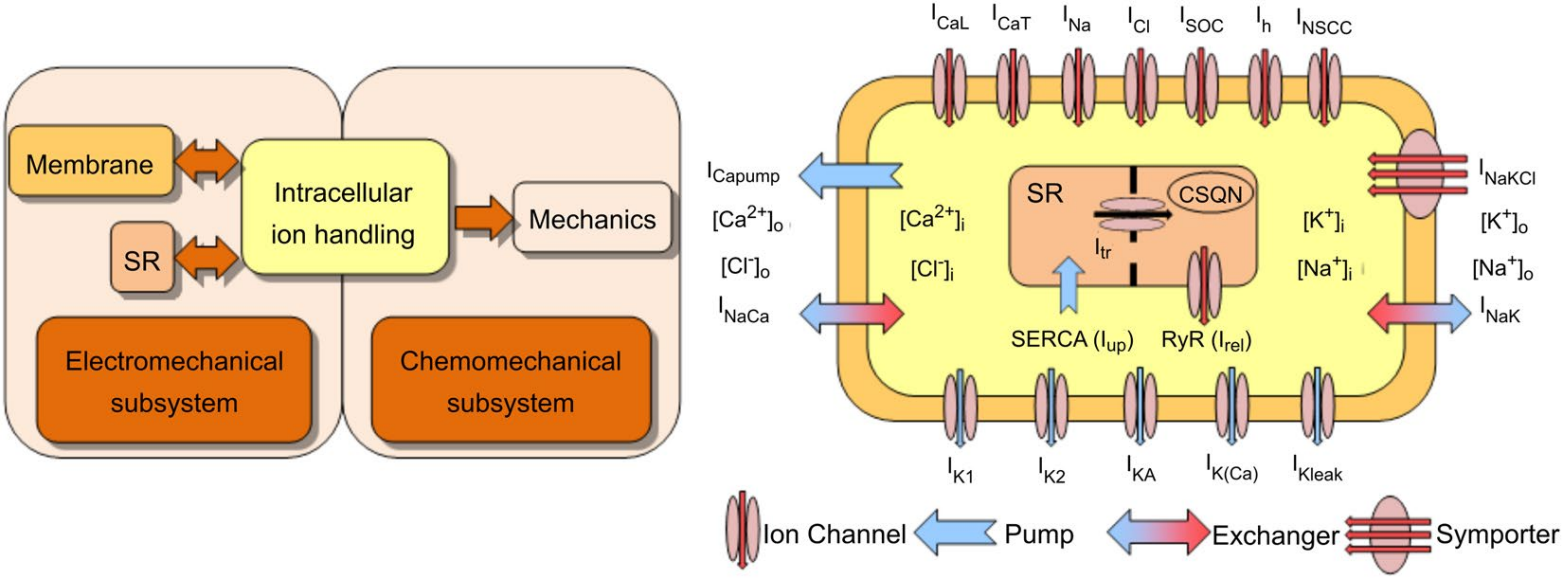
Schematic illustration of the myometrial cell model^49^. Left: The electrochemical and chemomechanical subsystems are coupled via the intracellular calcium concentration. The arrows indicate the direction of influence of the coupled systems. Right: Model components of 7 inward membrane channels (red arrows), 5 outward membrane channels (blue arrows), a calcium membrane pump, a Na-K-Cl symporter, 2 membrane ion exchangers, a SERCA pump (I_up_), an SR transfer current (I_tr_) and an SR release current (I_rel_) controlled by the ryanodine receptors (RyR) were shown. The colours in the flow diagram match their respective systems in the cell diagram.

To model the physiology of the single uterine cell, two distinct subsystems (electrochemical and chemomechanical) were coupled as shown in Fig. 1. The electrochemical subsystem is divided into the membrane (electrophysiology), intracellular ion handling and sarcoplasmic reticulum (SR) components. The electrochemical subsystem communicates with the chemo-mechanical subsystem using intracellular Ca^2+^ as a mediator. The mechanical force generation component handles the Ca^2+^ activation of contractile proteins and their regulation, and the subsequent production of tension.

The cell membrane contains ion channels, pumps and exchangers that permit the passage of charged ions (Fig. 1). These ionic channels are voltage-gated, but some are dependent on ionic concentrations. Typically a channel has activation gates and inactivation gates; each is described by a set of mathematical equations for describing steady states and time constants of the channel (Supplement A).

The Tong *et al*. model was used as the basal model, which was modified to incorporate detailed models of Ca^2+^ release from the sarcoplasmic reticulum (SR) and four-component force models, as developed by Yang *et al*.^18^, and the kinetic cross-bridge model developed by Hai and Murphy^19^ (details are provided in Supplement A).

### Cellular Electromechanical Activity

The myometrial cell is either at rest or exhibiting electrical and contractile activity. The rest state represents the quiescence exhibited by the cell between periods of activity; it is prevalent during most of gestation^20^. The active state represents the bursting activity that triggers contraction. Myocytes in the rat uterus are organised in two layers – the inner circular and outer longitudinal layer. Myometrial cells from the circular layer produce plateau-like action potentials (AP), and those from the longitudinal layer are spike-like^21^.

The action potential of the uterine single cell can also take various forms depending on the stage of pregnancy. Typically they are plateaux, spikes or a combination of the two. A successful labour is a sequence of contractions, and during each contraction there are rapid bursts of consecutive spikes that lead to the accumulation of intracellular Ca^2+^. Bengtsson *et al*.^22^ demonstrated AP can have variant morphologies in late pregnant rat myometrial cells from the circular layer. They begin as single spikes with plateaux. Over time the plateau potential begins to oscillate with increasing frequency and magnitude until it builds up to a rapid burst of spikes. Spike bursts increase in frequency and contractions increase in strength and duration until parturition^23^.

Gestational changes take place over the course of pregnancy. In non-pregnant rats uterine myocytes have a volume of ~2.5pL that increases 8-fold during pregnancy^9^. Spontaneous action potentials, typically last tens of seconds with a resting potential of ~−55 mV and a peak spike potential of ~10 mV^24^. During gestation the peak sodium current approximately doubles, while the peak calcium current halves^25^.

### General Model Equations

The total membrane ionic current density (I_tot_) is a sum of the currents from the various ion channels, pumps and exchangers. The change in membrane voltage is given by,

1 $$\begin{array}{rcl}\frac{dV}{dt} & = & -\frac{{I}_{tot}}{{C}_{m}}\\ {I}_{tot} & = & {I}_{h}+{I}_{CaL}+{I}_{CaT}+{I}_{Na}+{I}_{K1}+{I}_{K2}+{I}_{KA}+{I}_{KCa}+{I}_{NSCC}+{I}_{Cl}\\  &  & +\,{I}_{Capump}+{I}_{NaK}+0.5{I}_{NaCa}+{I}_{sus}+{I}_{soc}+{I}_{Kleak}+{I}_{NaKCl}\end{array}$$

where I_h_ is the hyperpolarisation-activated current, I_CaL_ the L-type calcium current, I_CaT_ the T-type calcium current, I_Na_ the fast sodium current, I_K1_ and I_k2_ the voltage-dependent potassium currents, I_KA_ the A-type transient potassium current, I_KCa_ the calcium-activated potassium current, I_NSCC_ the non-selective cation current, I_Cl_ the calcium-activated chloride current, I_Capump_ the calcium pump current, I_NaK_ the sodium/potassium exchanger current, I_NaCa_ the sodium/calcium exchanger current, I_sus_ the sustained background current, I_soc_ the store-operated non-selective cation current, I_Kleak_ the background potassium leakage current and I_NaKCl_ the sodium-potassium-chloride co-transport current. Current densities are given in units of pA pF^−1^ and specific membrane capacitance (C_m_) has a value of 1.4 µF cm^−2^ ^9^.

Details of the model equation development for each of the ion channel currents in Equation (1) are provided in the Supplementary Materials. The data for each ion channel can be found in Supplement A, the equations and parameters in Supplement B and the initial conditions in Supplement C. In simulations, Equation (1) is numerically solved using a forward Euler scheme with a time-step of 0.01 ms that provides stable solutions.

## Results

**Figure 2:**
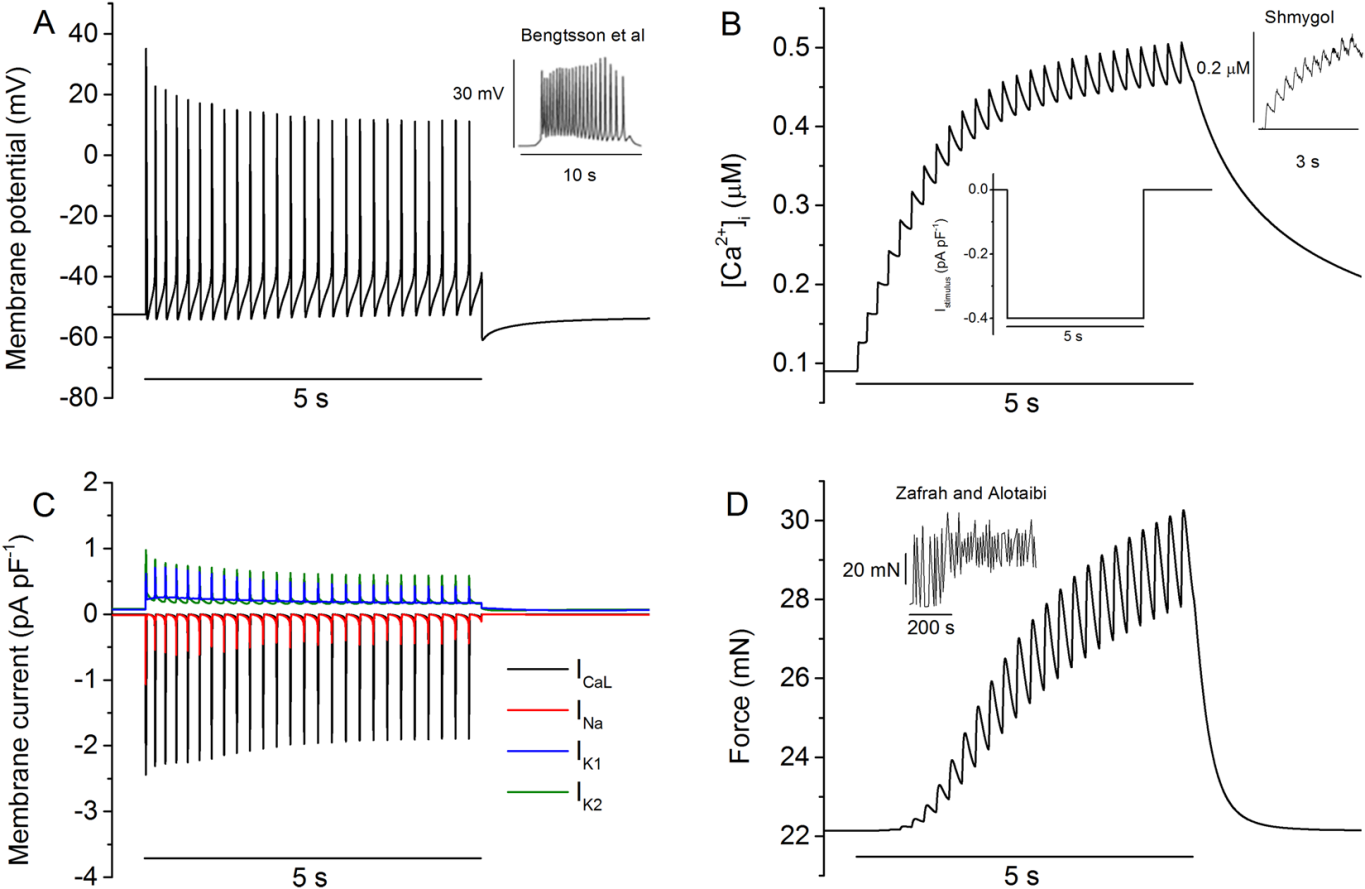
Bursting electrical activity of the model and comparison with experimental data. (**A**) Simulated bursting action A Inset: experimental data of bursting AP from Bengtsson *et al*.^22^. (**B**) Simulated staircase-like calcium transient generated by bursting APs. B Inset (centre): the stimulus applied during this simulation, −0.4 pA pF^−1^ for a duration of 5 s. B Inset (right): experimental data from Shmygol *et al*.^28^. (**C**) Computed results of four principal currents: L-type calcium, sodium and voltage gated potassium currents (I_K1_ and I_K2_) during the time course of bursting APs. (**D**) Simulated total contractile force of the cell model. D Inset: experimental data from Zafrah and Alotaibi^32^.

The developed model for the uterine myometrial cell is able to reproduce the typical electrical behaviours of the myometrial cell, including a stable resting potential in the absence of an external stimulus and bursting membrane potentials, which may be periodic and perhaps quasiperiodic and even chaotic in response to external stimuli. Figure 2 shows the simulated bursting action potentials in response to an external depolarising stimulus pulse, with a duration of 5 s and amplitude of −0.4 pA pF^−1^, which is <12% of the maximal inward ionic current density. It was shown that in response to such an external stimulus, a sequence of bursting action potentials were evoked (Fig. 2A) from a stable equilibrium resting potential, resulting in a stair-case increase of the intracellular Ca^2+^ concentration (Fig. 2B). Such bursting action potentials were generated by the integral action of opening and closing of many ion channels, some of which were shown in Fig. 2C. The simulated bursting action potentials well matched to those recorded experimentally. The contractile force of the membrane in response to the action potentials is shown in Fig. 2D. The simulated bursting action potentials, stair-case increase of the intracellular Ca^2+^ transient and the resultant cellular active force matched to examples of experimental data as shown by the insets in Fig. 2, which show the typical morphology of membrane potentials, calcium transients and force profiles in uterine cells from late-pregnant rat.

### The effects of AP morphology on [Ca^2+^]_i_

**Figure 3:**
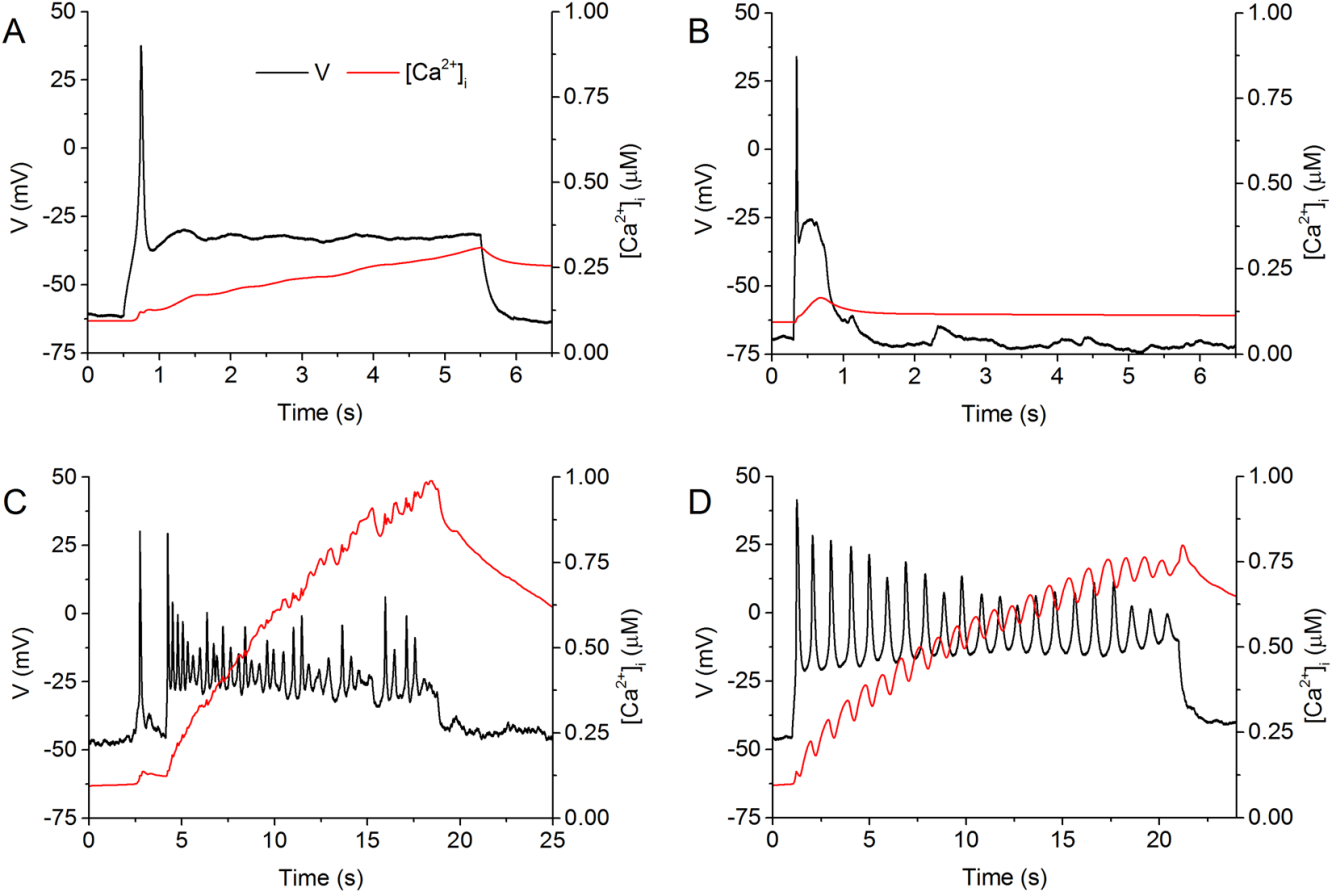
Simulated calcium transients produced by voltage clamps. The model voltage was clamped by using the voltage profiles obtained from experimental current clamp recordings, rather than constant values. The experimental data was recorded at 35 °C using the fluorescent indicator indo-1 in patch-clamped, single uterine myocytes from pregnant rats. A comprehensive methodology can be found in Shmygol *et al*.^28^. The parameters of each experimental current clamp are described in (**A**–**D**). (**A**) A single spike followed by a long plateau, using a holding current of −30 pA and a pulse of 60 pA for 5 s produced a short-lived, low maximum linear rise in [Ca^2+^]_i_. (**B)** A spontaneous single spike followed by a short plateau, using a holding current of −4 pA and no pulse produced a very short-lived spike in [Ca^2+^]_i_ followed by decay. (**C**) A spike train burst, using a holding current of −20 pA and a pulse of 60 pA for 20 s produced a regular stepped rise in [Ca^2+^]_i_. (**D**) A spontaneous spike train burst, using a holding current of −5 pA and no pulse produced a stepped, linear rise in [Ca^2+^]_i_.

Experimentally variant morphologies of the AP have been recorded from myometrial cells^22,26^. It has also been shown that AP morphology affects the intra-cellular Ca^2+^ transient^8,27^ and therefore the resultant force. To test the capability of the model in reproducing the effects of AP morphology on the intracellular [Ca^2+^]_i_, in simulations, we implemented variant experimentally measured membrane potentials recorded from freshly isolated 21 day pregnant rat myometrial cells (digitized at 2.5 kHz) as clamp commands to drive the cell model. Results are shown in Fig. 3, in which two distinct forms of action potentials were used: a single spike followed by either a smooth plateau or a train of spikes superimposed on the plateau. In responding to the AP clamp, the model produced variant forms of Ca^2+^ transient (red lines).

It was shown that the maximum [Ca^2+^]_i_ predicted by the model varies depending on the morphology of the AP. Of the various forms shown, high frequency action potential spike trains are the most effective way to build up the intracellular calcium to levels sufficient to initiate contraction over these long periods (Fig. 3C,D). These bursts tend to lead to a step-like morphology in the calcium transient. The 6 second plateau (Fig. 3A) produced a maximum [Ca^2+^]_i_ 2–3 times smaller than the 20 second bursts (Fig. 3C,D), and the 1 second plateau (Fig. 3B) produced half as much again. The simulated positive correlation between spike frequency and amplitude of [Ca^2+^]_i_ as shown in Table 2 qualitatively resemble those observed experimentally in the uterus^8,27^, further validating the model development.

**Table 2:** Properties of simulated [Ca^2+^]_i_ transient during voltage clamp.

Figure	τ_rise_ (s)	τ_decay_ (s)	[Ca^2+^]_i,max_ (µM)	Δ[Ca^2+^]_I_ (µM)
3A	4.86	5.69	0.31	0.21
3B	0.37	2.33	0.17	0.07
3C	6.76	13.85	0.99	0.89
3D	10.02	18.73	0.80	0.70

The minimum, maximum and increase in [Ca^2+^]_i_ in rat myometrial cells, including time constants describing the rise and decay of peak calcium.

**Figure 4:**
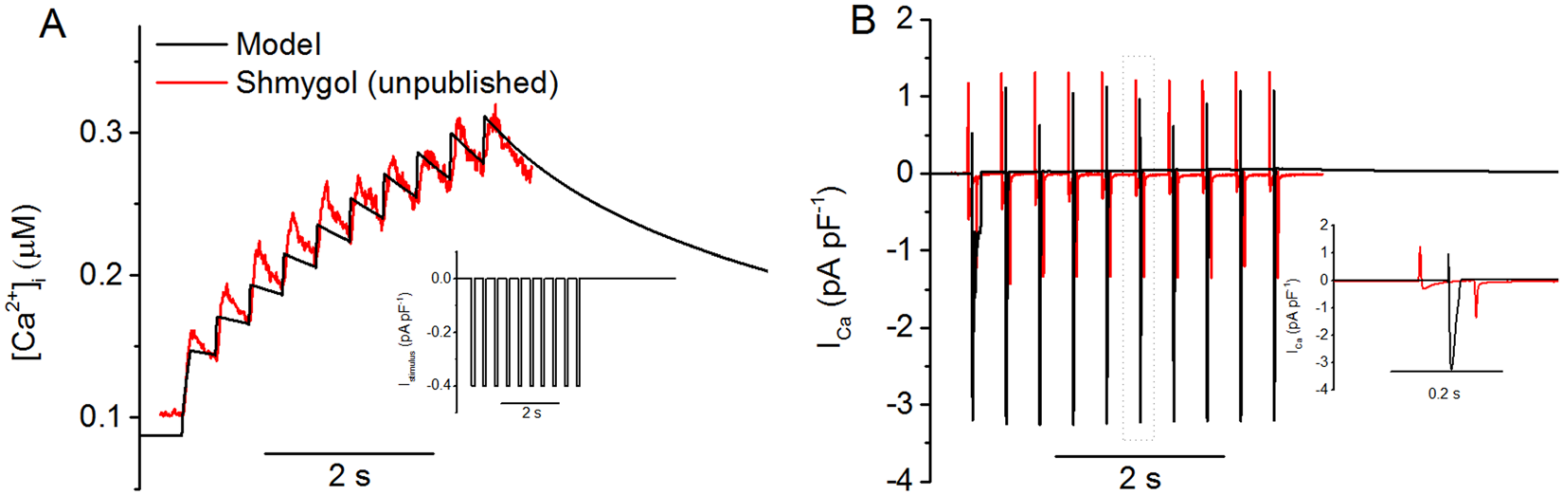
[Ca^2+^]i and I_Ca_ were observed under repetitive depolarising pulse train stimulations of −0.4 pA pF^−1^ at 2.5 Hz. Experimental results were obtained using a depolarising pulse of −80 to 0 mV at 2.5 Hz. The experimental data was recorded at 35 °C using the fluorescent indicator indo-1 in patch-clamped, single uterine myocytes from pregnant rats. A comprehensive methodology can be found in Shmygol *et al*.^28^. In order to reproduce the experimental data, the following model parameters from the standard model were multiplied by the factors shown: g_CaL_ × 0.9 and g_Na_ × 6. (**A**) Myometrial calcium transients exhibit a staircase-like increase. [Ca^2+^]_i_ ranges from 0.1 to 0.3 μM. A inset: the pulse train protocol. (**B**) The whole-cell calcium current recorded under voltage clamp shows a negative stair-case phenomenon. Note incompletely compensated capacitive currents. B Inset: a detailed view of the highlighted area.

Shmygol *et al*.^28^ demonstrated experimentally that repetitive depolarising current stimuli also produced a stair-case rise in intracellular Ca^2+^. This phenomenon is also re-produced by the model, and results are shown in Fig. 4, in which the simulated intracellular Ca^2+^ transient (Fig. 4A) and the underlying I_Ca_ (Fig. 4B) during the time course of repetitive stimulus pulses (inset in Fig. 4A) are shown. During the time course of repetitive current stimuli, the simulated stair-case rises in [Ca^2+^]_i_ matched to experimental data (Fig. 4A), however, the time course of I_Ca_ shows notable difference in the amplitude of I_Ca_ between simulation and experimental data. In both model and experimental data, the total calcium current exhibits a spike-like behaviour switching between positive and negative spikes. However, the model’s current magnitudes were skewed into the negative compared with experimental data, having an inward current magnitude of approximately double. Note that I_Ca_ does not flip between an inward and outward direction. Experimentally the switching spikes are a result of capacitive currents that were not fully compensated for the cell’s capacitance exceeded 100 pF, whereas the amplifier’s range of compensation is limited to 100 pF. In the model the positive spikes are a result of the stimulus pulse causing large depolarising spikes that exceed the calcium channel reversal potentials. Such differences explain the discrepancy between the experimental results and the model in I_Ca_ morphology as shown in Fig. 4B. Table 3 shows a range of frequency and magnitude values for I_Ca_, and our simulation data are qualitatively similar to experimental data.

**Table 3:** Properties of simulated I_Ca_ under repetitive depolarising pulse train stimulations of −0.4 pA pF^−1^ at variant stimulation rates.

f (Hz)	I_Ca,in_ (pA pF^−1^)	I_Ca,out_ (pA pF^−1^)	ΔI_Ca_ (pA pF^−1^)	Ref
500	2.47	0.00	2.47	^9^
20	3.00	0.00	3.00	^10^
6.7	5.00	0.00	5.00	^36^
3	0.50	0.10	0.60	^28^
2.5	1.50	1.25	2.75	Shmygol
2.5	3.20	1.10	4.30	Model (Fig. 4B)

The frequency, magnitude of the inward/outward calcium currents and the range of peak current magnitudes in rat myometrial cells.

During the repetitive pulse stimulation, the characteristics of the simulated stair-case rise of [Ca^2+^]_i_ matched to experimental data, which are summarised in Table 4.

**Table 4:** Comparison of the properties of [Ca^2+^]_i_ transients between simulated and experimental data under repetitive depolarising pulse train stimulations of −0.4 pA pF^−1^ at 2.5 Hz.

τ_rise_ (s)	τ_decay_ (s)	[Ca^2+^]_i,min_ (µM)	[Ca^2+^]_i,max_ (µM)	Δ[Ca^2+^]_I_ (µM)	Ref
70	162.20	0.13	0.18	0.05	^47^
2.2–4.5	11.7–24.8	0.10	0.60	0.50	^48^
3.4	2.90	0.10	0.32	0.22	Shmygol
2.9	6.80	0.08	0.31	0.23	Model (Fig. 4A)

The minimum, maximum and increase in [Ca^2+^]_i_ in rat myometrial cells, including time constants describing the rise and decay of peak calcium.

In the model, the Ca^2+^ transient has a τ_rise_ of 2.9 s, a τ_decay_ of 6.8 s and a magnitude of 0.23 µM (Fig. 4A), which is in close agreement with the data provided by A. Shymgol, which has respective values of 3.4 s, 6.9 s and 0.22 µM. The model’s I_Ca_ had a frequency of 2.5 Hz and a magnitude range of 4.3 pA pF^−1^ (Fig. 4B). This is also in good agreement with the data, which has respective values of 2.5 Hz and 2.75 pA pF^−1^. All of these show a close match between simulation and experimental data, which further validated the model development.

### Modelling drug effects

In order to further validate the developed model, further simulations were conducted to examine if the model could reproduce the action of a variety of hormone and drugs on modulating the electrophysiological action potential, calcium handling and force production.

#### Simulated effects of oestradiol

Previous studies have shown that in the presence of oestradiol, spontaneous AP bursts and muscle contractions were suppressed^26,29^. Further studies have shown that the suppressive action of oestradiol may be attributable to its inhibitive actions on the voltage-dependent calcium, calcium-dependent potassium and voltage-dependent potassium channel currents, contributing to the prevention of the genesis of spiking AP^29,30^. To test the ability of the model to reproduce the effect of oestradiol, the action of oestradiol on its targeted membrane ion channels was simulated by applying the conditions shown in Table 5 and comparing the response to the experimental data of Inoue *et al*.^26^.

**Table 5:** The effects of oestradiol are simulated by scaling down the conductance of the above channels.

**Calcium Currents (Voltage-Operated)**	
g_CaL_	0.2
**Potassium Currents (Voltage-Operated)**	
g_K1_	0.6
g_K2_	0.6
**Potassium Currents (Calcium-Operated)**	
g_K(Ca)_	0.9

These values were derived empirically by matching the resulting morphological changes to experimental findings and are multiplying factors applied to the conductances of the standard model described in the appendices.

**Figure 5:**
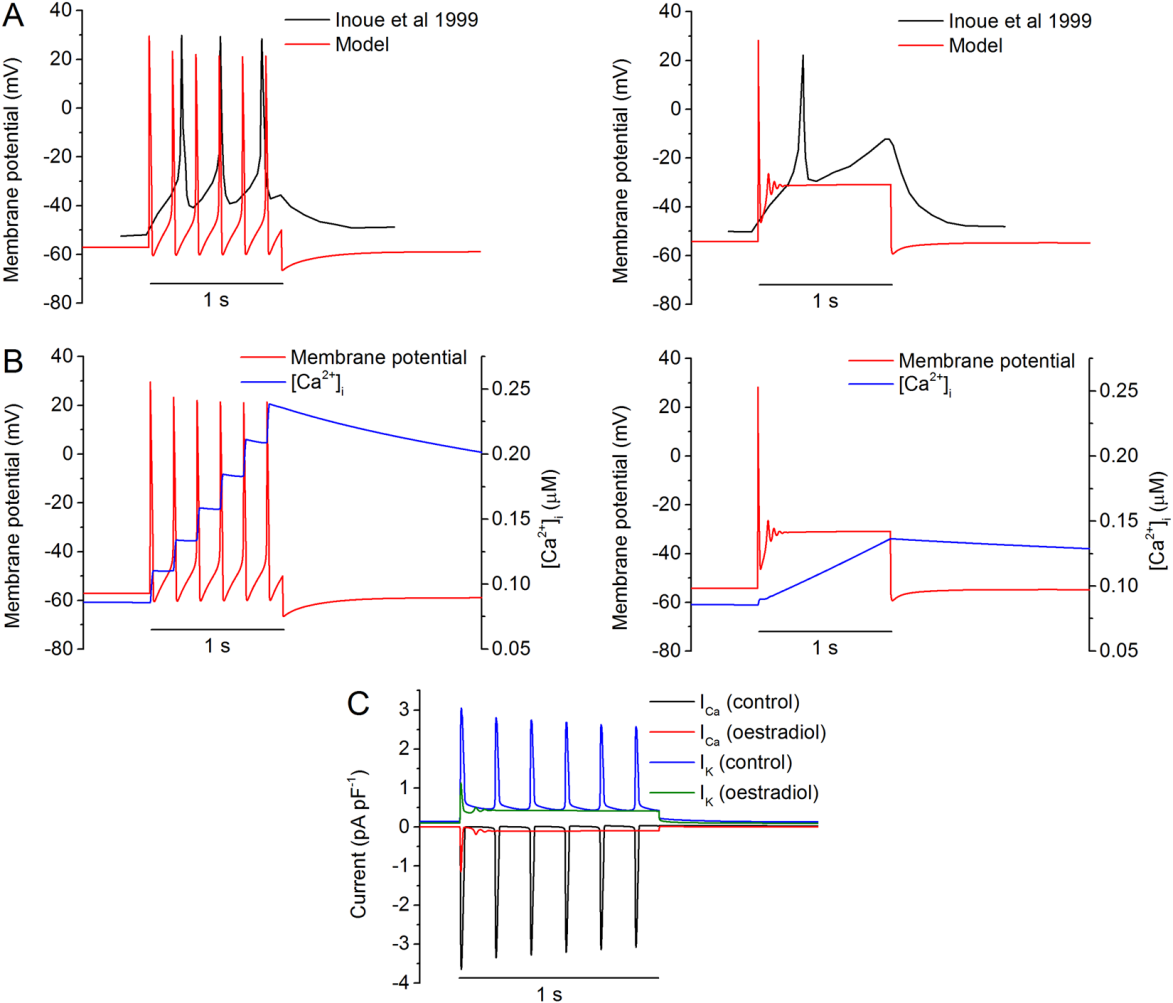
Simulated effects of oestradiol. (**A**) Experimental (black) and simulated (red) membrane potentials under control (left) and oestradiol (right) conditions. Experimentally a 1 s stimulus of 20 pA was applied, with or without 30 µM of oestradiol. In simulations a stimulus current of −0.4 pA/pF was applied to the cell for 1 s. Oestradiol was applied by altering channel conductances (Table 5). (**B**) Influence of oestradiol on simulated intracellular calcium levels (blue), under control (left) and oestradiol (right) conditions. (**C**) Effects of oestradiol on total calcium and potassium currents.

Figure 5 shows the simulated spiking APs (Fig. 5A) and the underlying [Ca^2+^]_i_ (Fig. 5B) in the control (left panels) and oestradiol (right panels) conditions in response to a stimulus current of −0.4 pA pF^−1^ applied to the model. In the control condition, such a stimulus generated a sequence of spiking APs that were quantitatively similar to the experimental data from Inoue *et al*. (Fig. 5A), which had a resting potential of ~−50 mV and peak of ~30 mV, with a frequency of ~4 Hz. In response to such repetitive spiking APs, the simulated intracellular Ca^2+^ concentration accumulated, generated a stair-case trace of [Ca^2+^]_i_ (Fig. 5B). In the presence of oestradiol, the spiked APs were supressed, resulting in an AP with a long plateau phase. In such a case, the intracellular Ca^2+^ concentration accumulated to a lesser extent, resulting in a smaller [Ca^2+^]_i_ amplitude (about 43% reduction in peak calcium) as compared to the control condition. Note that in the presence of oestradiol, the usual staircase-like increase in calcium instead became a smooth, linear increase.

The underlying ion channel currents of I_Ca_ and I_K_ in control and oestradiol conditions are shown in Fig. 5C. Okabe *et al*. demonstrated that oestradiol has a similar effect on both the total calcium and total potassium currents^29^; they reported a difference in reduction of I_Ca,max_ and I_K,max_ of 4.4% to 8.4%, with I_Ca,max_ undergoing the greater drop. Simulated currents show a difference of 8%, with a greater fall in I_Ca,max_ than I_K,max_ (Fig. 5C).

The simulated depressive effects of oestradiol matched to experimental observations. This in one hand validated the model by demonstrating the capability of the model to reproduce experimental observations of oestradiol. On the other hand the simulation results also provided mechanistic insights into understanding the depressive actions of oestradiol, which are attributable to the integral action of oestradiol on I_CaL_, I_K1_, I_K2_ and I_K(Ca)_.

### Simulated effects of oxytocin

The myometrium possesses specific receptors for oxytocin, which is a hormone used to induce labour. It has been shown that oxytocin promotes contraction by increasing [Ca^2+^]_i_ and is up-regulated on the approach to labour^31^. Studies have also shown it increases both the frequency and amplitude of contractions in rat uteri^32–35^.

The mechanism underlying the promoting contraction of oxytocin is incompletely understood. In their study Inoue *et al*. demonstrated that oxytocin increases the calcium channel currents in rat myometrium, but has little to no effect on the sodium and delayed-rectifier potassium channels, unlike in rabbit where it’s effect on sodium channels is important^36^. It is unclear if the observed increases in the calcium channel currents can produce the promoting action of oxytocin.

**Figure 6:**
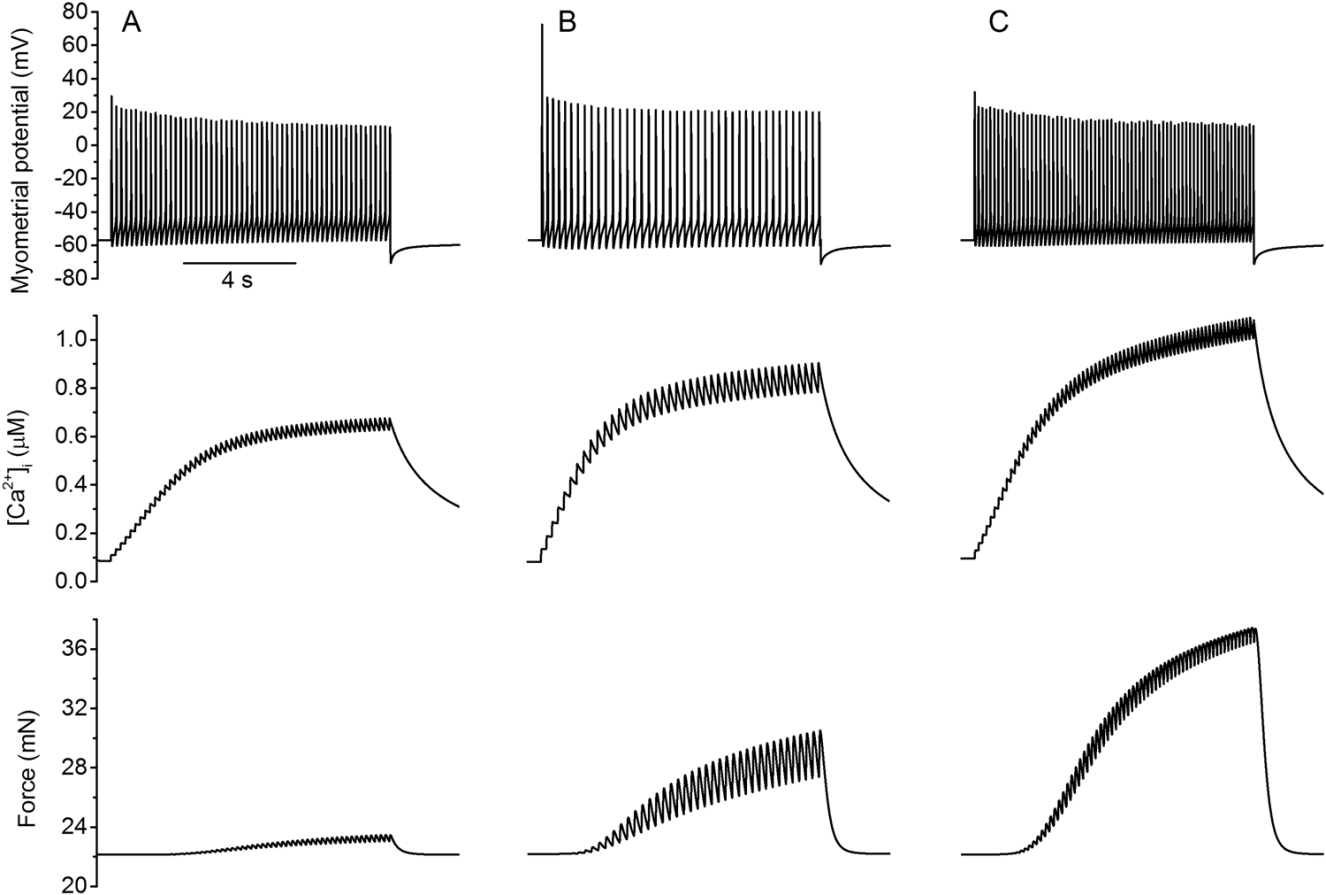
Simulated effects of oxytocin on the membrane potential (top panels), calcium transient (middle panels) and force profile (bottom panels) of a rat uterine cell. A 10 s stimulus of −0.4 pA pF^−1^ was applied to the membrane, corresponding to a control dose of oxytocin at ~1 nM. (**A**) Control condition using the standard model. (**B**) L-type calcium was upregulated by adjusting its conductance by a factor of 1.8. (**C**) T-type calcium was upregulated by a factor of 10.

In simulations, we investigated the role of an increased I_CaL_ or I_CaT_ on the electrical and mechanical behaviours of the myometrial cell model. Results are shown in Fig. 6 for control (Fig. 6A) and increased I_CaL_ (Fig. 6B) and I_CaT_ (Fig. 6C). It was shown that up-regulating L-type calcium increases intracellular calcium concentration by 38%, and consequently contractile force by 30%. However it reduces the frequency of the driving action potential by 26%, resulting in less frequent contractions (Table 6).

**Table 6:** Simulated effects of oxytocin.

Condition	f (Hz)	τ_rise_ (s)	τ_decay_ (s)	[Ca^2+^]_i,max_ (µM)	Δ[Ca^2+^]_i_ (µM)	F_max_ (mN)
Control	5.7	4.67	12.68	0.68	0.60	23.45
L-type up	4.2	4.11	10.13	0.91	0.83	30.55
T-type up	7.1	3.82	9.16	1.10	1.02	37.46

The frequency of the membrane potential, rise time, fall time, peak value and change in [Ca^2+^]_i_ and maximum contractile force when up-regulating L-type and T-type calcium channels by factors of 1.8 and 10, respectively.

Alternatively, up-regulating T-type calcium increased both the frequency of contractions by 25% and [Ca^2+^]_i,max_ by 62%. This more closely represents the previous experimental observations^32–35^ and suggests that I_CaT_ may play a more significant role than I_CaL_ in the action of oxytocin on rat myometrium. This resembles the behaviour invoked by sodium channel up-regulation in other species and may be explained by the T-type calcium activation (Fig. A2C) being approximately twice as fast as the L-type (Fig. A1C) in the model. The sodium activation is ~8 times as fast as the L-type calcium (Fig. A3C).

### Simulated effects of nifedipine

Nifedipine is an L-type calcium blocking agent used to treat pre-term labour, as well as a variety of other conditions relating to muscle contraction or spasms. It is a commonly used tocolytic (anti-contraction medication) favoured for its few side-effects^37^. It is cheap, given orally and significantly decreases the risk of delivery within 7 days of commencing treatment^38^. Reports state that the application of nifedipine can reduce the level of intracellular calcium or even eliminate it entirely^28,39–41^.

**Figure 7:**
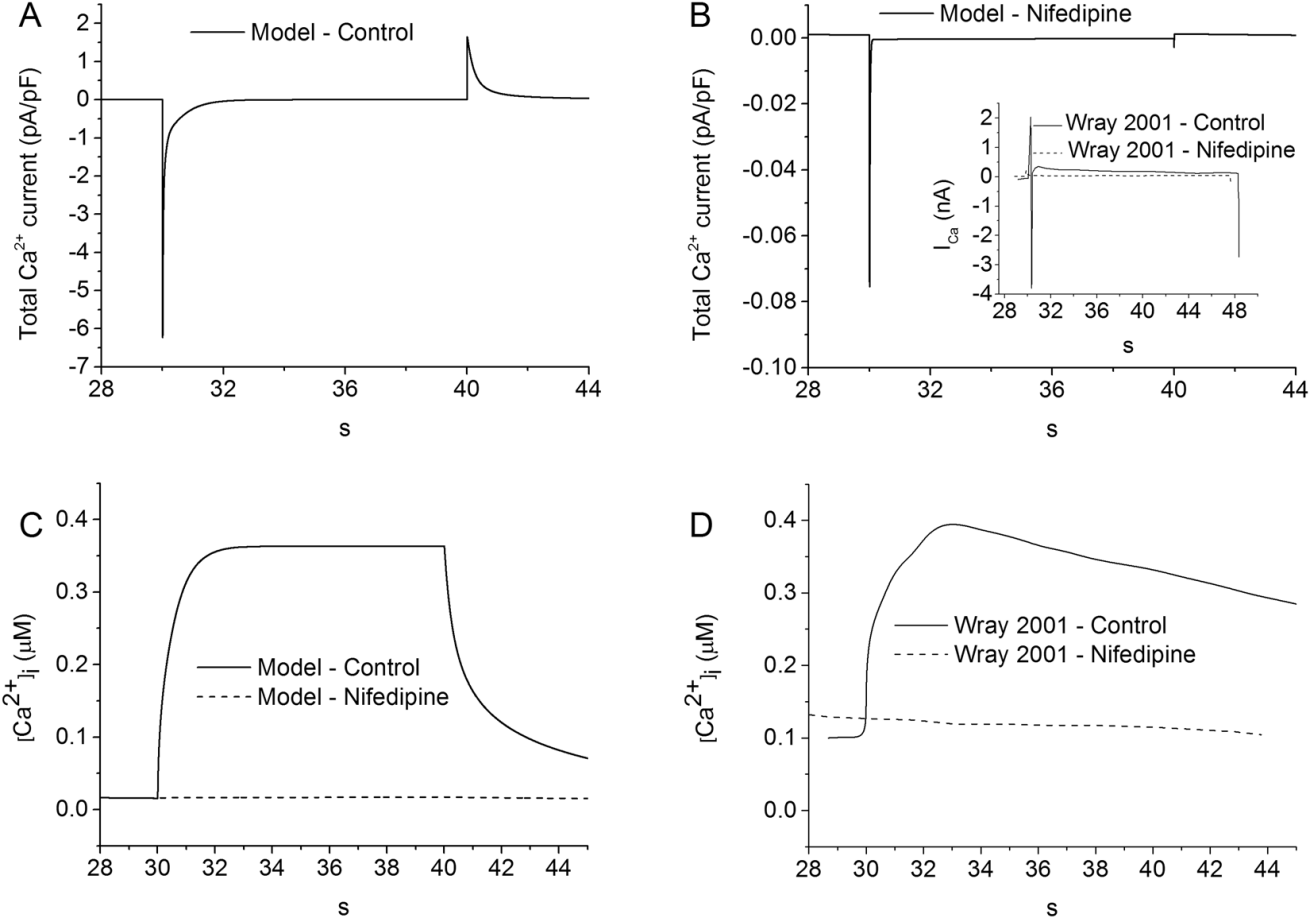
The influence of 10 µM of nifedipine on the total calcium current and Ca^2+^ transients. A voltage clamp from −80 to 0 mV was applied to the cell membrane for 10 seconds. (**A**,**B**) The membrane voltage response to the control and nifedipine conditions respectively, showing a drop of approximately 100 times in the maximum current value. B (inset): Experimental data from Wray *et al*. demonstrating the drop in current due to the action of nifedipine^42^. Both simulations (**C**) and experiments (Wray *et al*.^42^) (**D**) suggest that in the presence of nifedipine, applying a voltage across the membrane prevents an influx of calcium into the cell.

In order to further validate the model, effects of blocking I_CaL_ on the intracellular Ca^2+^ concentration were simulated in the condition of a voltage clamp, from −80 to 0 mV for 10 s. Results are shown in Fig. 7. In simulations, blocking I_CaL_ decreases the amplitude of total I_Ca_ by approximately 98.7% (Fig. 7A,B), and almost abolished the intracellular Ca^2+^ transient (Fig. 7C), which closely matched the experimental observation of Wray *et al*.^42^ (Fig. 7D).

**Figure 8:**
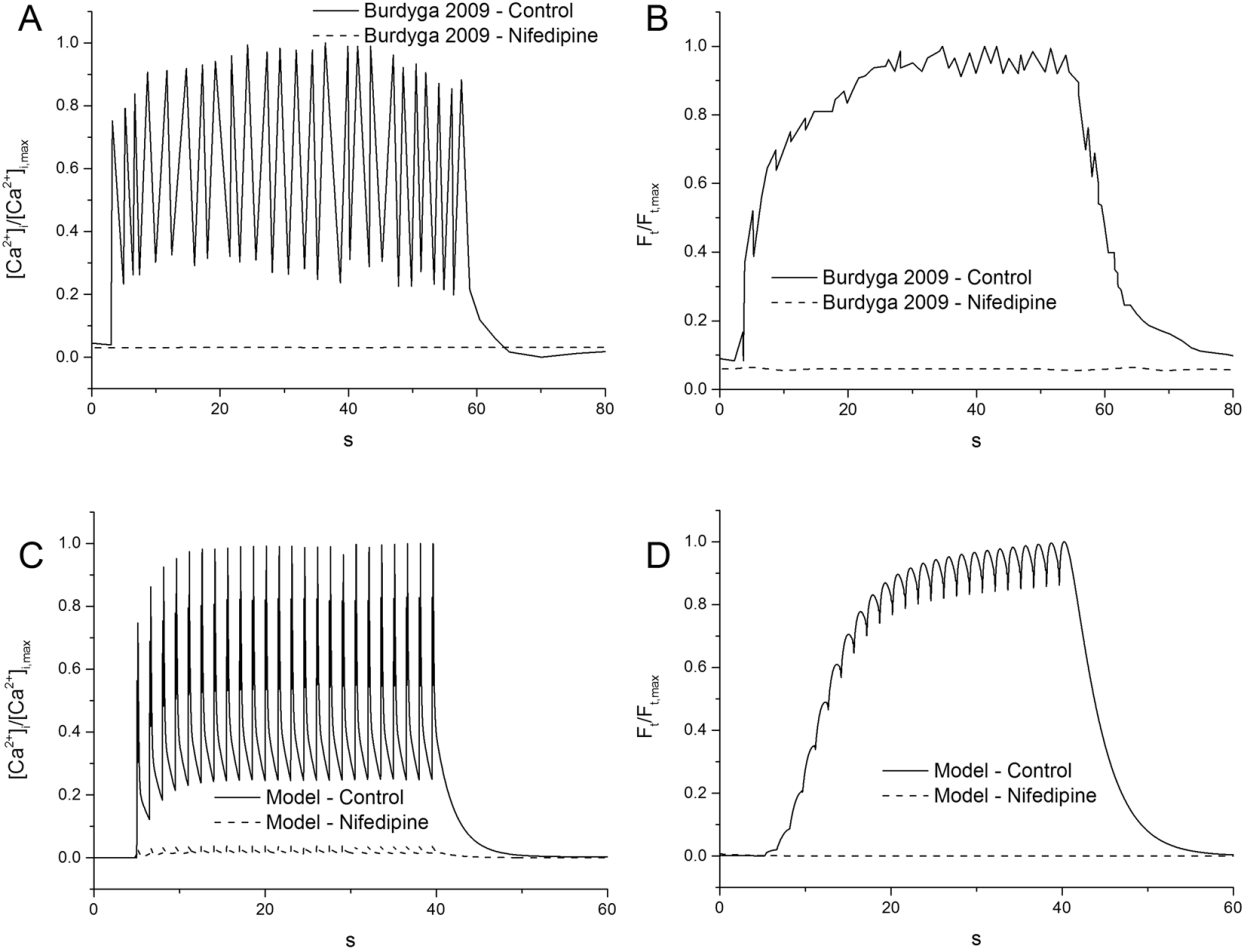
The effect of 10 µM of Nifedipine on [Ca^2+^]i and the contractile force of the single uterine cell. The model results were obtained by applying a current pulse of −0.7 pA pF^−1^ for 0.2 s every 1.5 seconds for 40 seconds. The experimental data was collected from tissue samples by Burdyga *et al*.^7^. Each dataset was normalised between its zero and one to allow a comparison of the morphology. (**A**,**B**) Experimental levels of intracellular calcium and normalised force measurements, respectively. (**C**,**D**) Simulated levels of intracellular calcium and normalised force measurements, respectively. In the control case a rapid increase in Ca^2+^ is followed by spike-like oscillations, ending in a smooth drop. Calcium accumulation was suppressed by the presence of nifedipine. Nifedipine was simulated by applying a 95% block to the L-type calcium channels.

Further simulations were also conducted to investigate the effect of I_CaL_ blocking on the membrane APs and contractile force of the cell model by applying a current pulse of −0.7 pA pF^−1^ for 0.2 s every 1.5 seconds for 40 seconds. The results are shown in Fig. 8. It was shown that blocking I_CaL_ abolished cell APs (Fig. 8A), resulting in a small contractile force (Fig. 8B), which closely matched the experimental observation of Burdyga *et al*., who demonstrated that blocking the main entrance for calcium into the cell with nifedipine has the effect of minimising the contractile stress across the membrane (Fig. 8C,D).

With a well validated model, we theoretically explored the possible roles of altered Na^+^ and Ca^2+^ channel currents in modulating cellular actions potentials.

**Figure 9:**
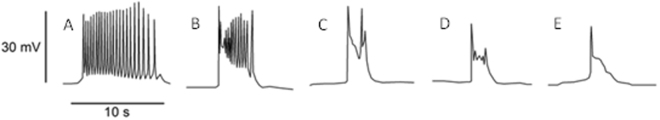
Characteristic spontaneous action potential waveforms recorded at various stages of gestation by Bengtsson *et al*.^22^, taken from rat circular muscle tissue at body temperature. Gestational age in days: (**A**) late 21, (**B**) early 21, (**C**) 20, (**D**) 18–19, and (**E**) 16–17. The chronology was reversed to more easily compare the change of morphology due to gestation with the effects of channel blocking in Figs 10 and 11.

**Figure 10:**
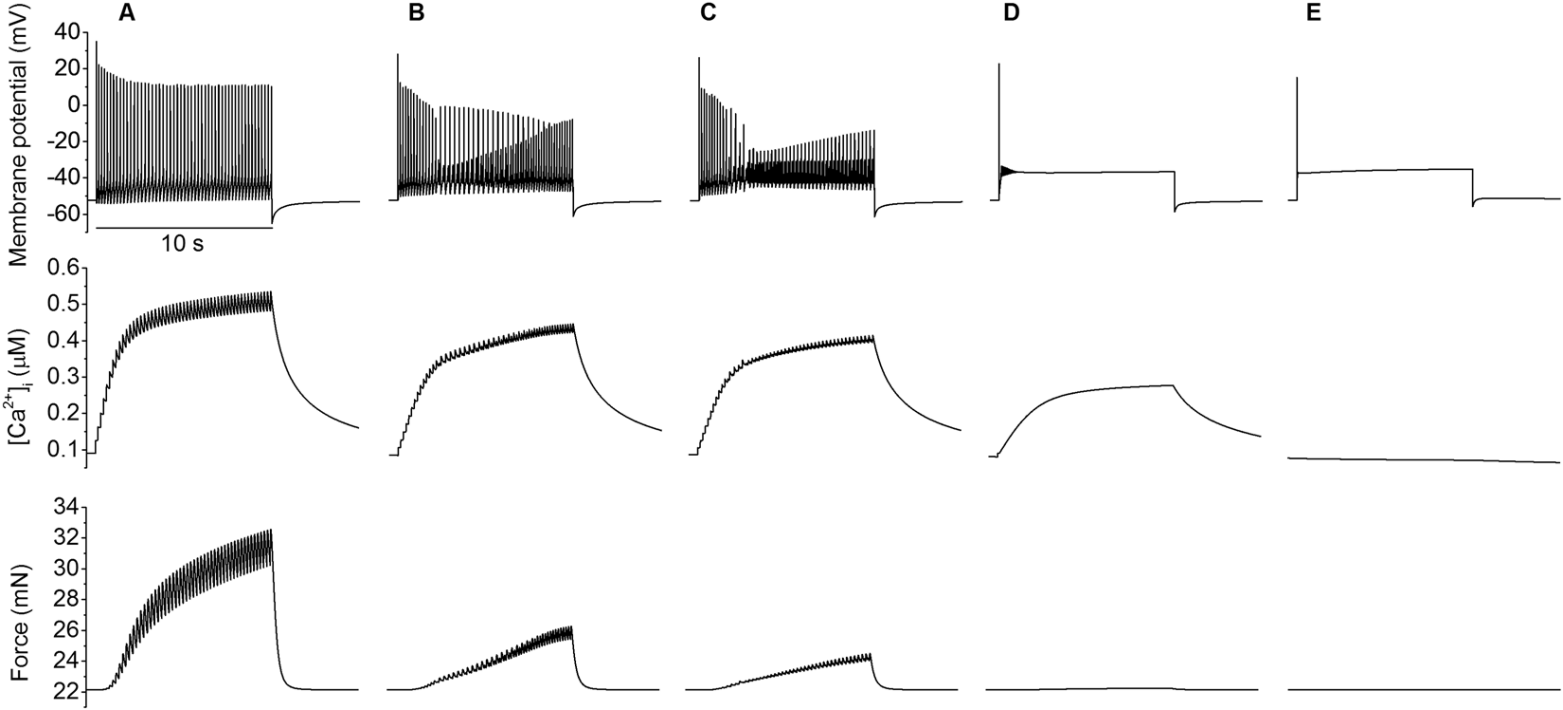
Effects of blocking L-type calcium channels on the AP and excitation contraction coupling. Action potentials (top panels), calcium transients (middle panels) and force profiles (bottom panels) produced by altering the maximum conductance of the L-type calcium current in the model. From left to right the conductance of g_CaL_ (**A**) 100%, (**B**) 62%, (**C**) 57%, (**D**) 33% and (**E**) 0% with a 10 second stimulus of −0.4 pA/pF applied.

Experimentally variant action potentials have been recorded from uterine myocytes during different stages of gestation as shown in Fig. 9. Such varied profile characteristics in membrane action potentials may be due to different levels of ion channel expression, which may go through a process of remodelling during the pregnancy^22^. To test this hypothesis, in Fig. 10, the effect of varied maximal channel conductance of the L-type calcium current g_CaL_ on membrane potential (top), intracellular Ca^2+^ concentration (middle) and the resultant force (bottom) were simulated. It was shown that under the condition of a current pulse stimulus–reducing g_CaL_ from 0.12 nS pF^−1^ to 0 (i.e. mimicking block of the channels or down-regulation of the channel) changed the response of the cell model from a burst of spikes to a single spike and plateau, in a sequence that reflects the changes in action potential shape observed during gestation (Fig. 9). Correspondingly, the time trace of the intracellular Ca^2+^ concentration and the resultant force also showed different profiles and amplitudes.

**Figure 11:**
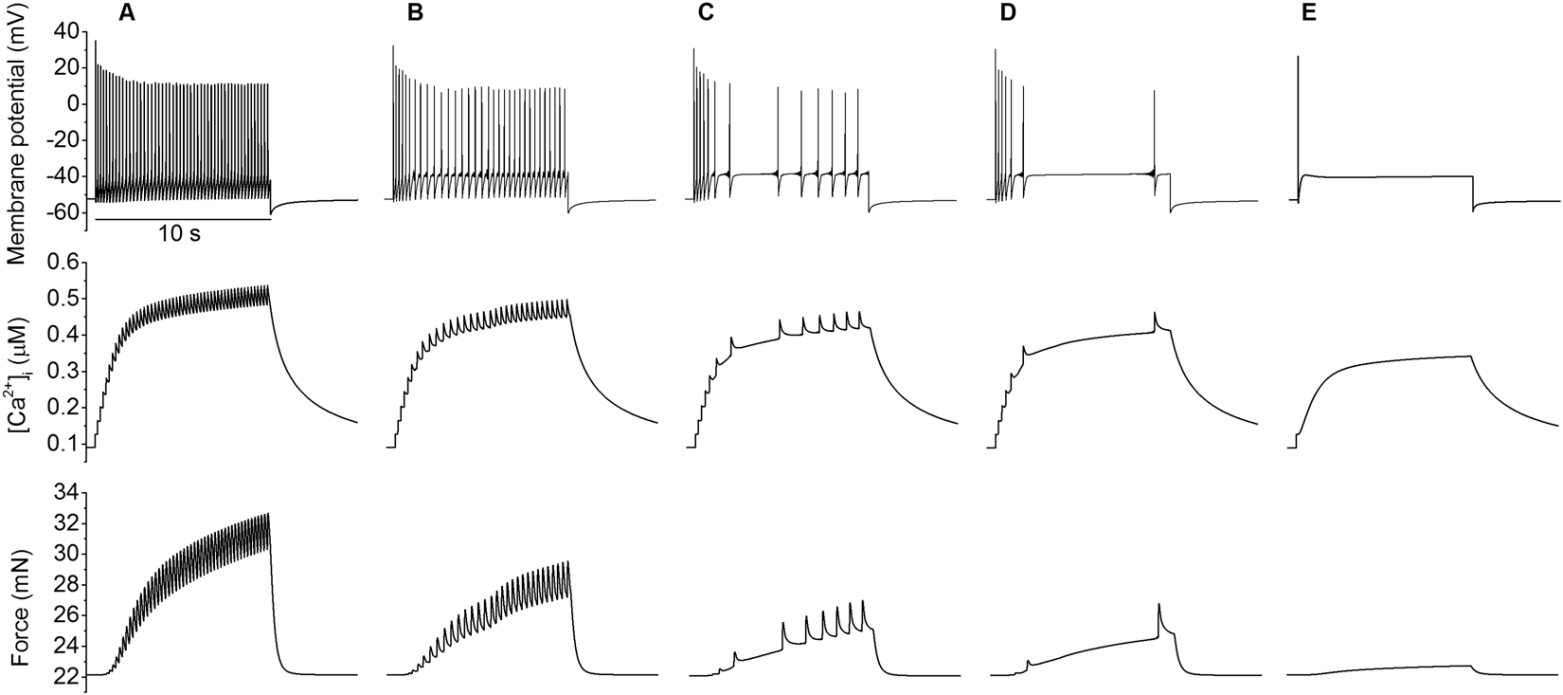
Effects of blocking fast sodium channels on the AP and excitation contraction coupling. Action potentials, (top panels), calcium transients (middle panels) and force profiles (bottom panels) produced by altering the maximum conductance of the fast sodium current in the model. From left to right the conductance of g_Na_ (**A**) 100%, (**B**) 74%, (**C**) 61%, (**D**) 57% and (**E**) 0% with a 10 second stimulus of −0.4 pA/pF applied.

Similarly, the profile of membrane action potentials is also dependent on the maximal conductance of the sodium current g_Na_. Figure 11 shows that a reduction in g_Na_ reduced the number of spikes evoked by the injected current pulse. As g_Na_ was reduced the discharge rate reduced from 5/s to a solitary action potential for reductions of >77%, with a minimum maintained discharge rate of 1.4/s at a reduction of 37%. Each spike was accompanied by an increase in [Ca^2+^]_i_, and during repetitive spiking these summate. At the end of a burst of spikes the [Ca^2+^]_i_, fell with a time constant of ~5.6 s.

Through the mechanism of electrical and mechanical coupling, changes in [Ca^2+^]_i_ were expected to be reflected in the resultant contraction force. If L-type calcium channel expression increases as pregnancy progresses then we would expect to see a gradual increase in the levels of intracellular calcium and consequentially a rise in contractile force. Lowering the conductance of the sodium channels had a greater effect on the frequency and amplitude of individual [Ca^2+^]_i_ and force spikes than the overall amplitude of their plateaus (Fig. 11), whereas lowering calcium channel conductance to 33% was enough to half [Ca^2+^]_i_ (Fig. 10).

**Figure 12:**
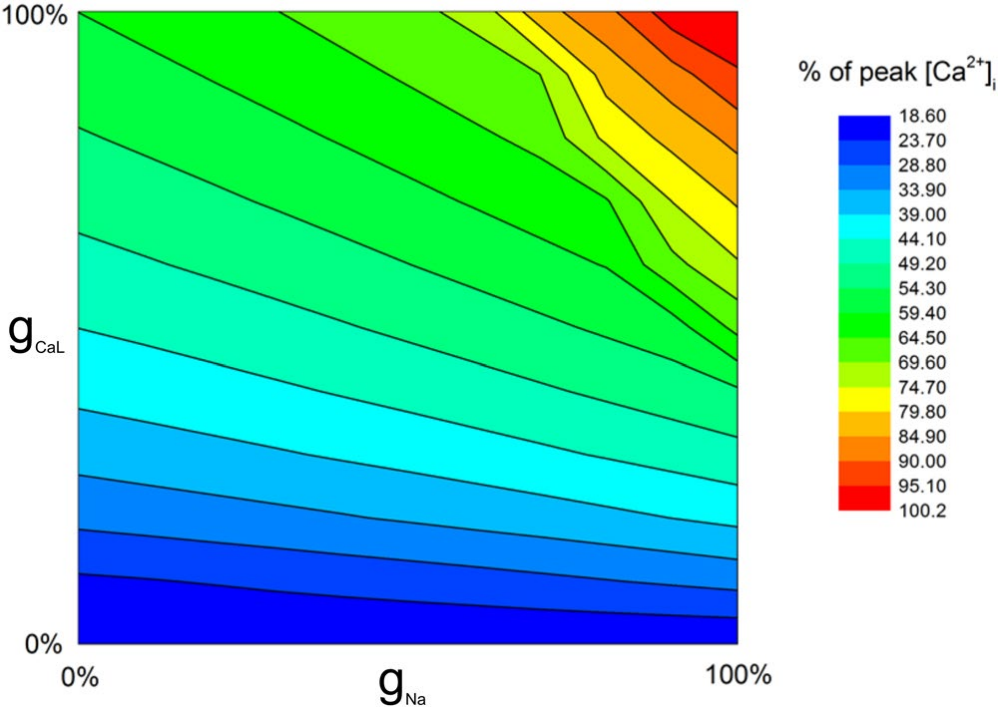
The gCaL – gNa parameter-space. The L-type calcium and fast sodium conductances were varied simultaneously in increments of 10% from 0% to 100% to assess the dependence of the [Ca^2+^]_i_ on the respective channels. The colour map indicates the concentration of peak calcium, relative to the maximum value where both conductances were set to 100%.

Figure 12 maps out the effect of simultaneous alterations of g_CaL_ and g_Na_ on the intracellular Ca^2+^ concentration. In the g_CaL_-g_Na_ parameter space, changing the g_CaL_ and g_Na_ parameters from the bottom left hand corner to the top right resulted in an increase in [Ca^2+^]_i_, which is analogous to the increased force shown over the course of gestation^43,44^. A variant combination of changes in the g_CaL_ and g_Na_ parameters affects the rate of change of cell activity. Increasing sodium channel density had a smaller effect on [Ca^2+^]_i_ than L-type calcium channel density until both reached ~50%. Changes in g_CaL_ produced a smooth increase throughout the map, whereas increases to g_Na_ in this top-right region resulted in a rapid change. For example, when g_CaL_ is set to 100% increasing g_Na_ from 0% to 50% results in a 10% increase in [Ca^2+^]_i_. Increasing it from 50% to 100% results in a larger increase in [Ca^2+^]_i_ of 30%. This may indicate that an increase in sodium channel density in late pregnancy could produce a phase change in behaviour leading to contractions.

## Discussion

In this study we present an updated model of the electrical, electro-chemical and mechanical activities of uterine smooth muscle cells based on modifications of the Tong *et al*. model^13^. The developed model was validated by its ability to reproduce variant action potential profiles, intracellular Ca^2+^ transients and generation of the active force, as well as the actions of some pharmacological agents, including oestradiol, oxytocin, nifedipine and wortmannin.

As compared to the previous Tong *et al*. model, the major advances of the present model are: (i) updated models for some of the membrane currents. The model considers membrane currents of I_CaL_, I_CaT_, I_Na_, I_K1_, I_K2_, I_KA_, I_h_, I_NaCa_, I_NaK_, I_NaKCl_, I_Cl_, I_SOC_, I_NSCC_, I_K(Ca)_ I_Ca-pump_ and I_K-leak_. Among them, model equations and parameters for ionic channel currents of I_CaL_, I_K2_, I_K(Ca)_ and I_Cl(Ca)_ were updated to fit more closely to experimental data; (ii) a more detailed description of the calcium handling system that consists of an SR model to describe calcium sequestration, uptake and release with three currents (SERCA, RyR and a transfer current) and a calcium buffer; and (iii) a more detailed description of cellular force generation. The mechanical model consists of a 4-state kinetic cross-bridge model that describes the cycling between phosphorylated/non-phosphorylated and attached/unattached states, and a 4-component Hill model with passive, active, cross-bridge elastic and visco-elastic forces. As summarised in Table 1, these advances form the most comprehensive rat uterine smooth muscle model, representing a significant improvement as compared to other models for simulating the electrical and mechanical activities of the uterine smooth muscle cells. In addition, the presented model has been comprehensively validated by comparison of simulation data to experimental findings (Figs 2–5, 7 and 8) of the profiles of action potentials, intracellular Ca^2+^ transient and force generation in control and in drug actions.

Using the model, we investigated possible mechanisms underlying the transition from a stable steady state to genesis of spontaneous electrical bursting activities during the gestational period. In the absence of external stimuli, the model has a robust and quiescent stable steady state. However, with increased sodium and calcium channel density, the model may show spontaneous bursting activity. In simulations, it was shown that an increase in the sodium channel density appears to be a major factor in changing the model from a quiescent state to a spontaneous bursting state, a main feature of the electrical activity of the uterus in labour (Fig. 12). There is a threshold over which a myometrial cell becomes more sensitive to up-regulation in sodium channel activity, so small changes in sodium channel density would cause significant qualitative changes in cell electrical behaviour. It was also shown that both sodium and calcium channel density play a role in determining the morphology of the action potential and the calcium transient (Figs 10 and 11). While the calcium channel current is more important in determining the amplitude of the calcium transient, the sodium channel current, however, is more important in determining the frequency of AP spikes. This indicates that a combination of changes in the two channels is required to produce the rapid bursting activity associated with the large accumulation of calcium needed for strong, long lasting contractions. These simulation results imply increases in sodium and calcium channel density may happen as a natural process of gestation over time.

The developed model was also used to investigate electrical and mechanical activity of the uterus in various physiological and pharmacological conditions. By altering selected parameters it can reproduce a variety of physiological and pathological behaviours, such as drug effects via pore block or environmental conditions by altering the extracellular ion concentrations. In simulations, the effects of four drugs and hormones on the electromechanical activities of the model were examined and compared to experimental findings for validation purposes. The simulation results for oestradiol (Fig. 5), oxytocin (Fig. 6) and nifedipine (Figs 7 and 8) all closely matched to experimental data. Our results indicate that that action of oestradiol on myometrial activity is attributable to its integral action on the reduction of conductance in multiple channels, including I_CaL_, I_K1_, I_K2_ and I_K(Ca)_; and the action of oxytocin may be attributable to the increased conductance of I_CaT_. Our results compare favourably to the effects of these drugs seen in the literature, suggesting the developed model may be used as a platform supplementary to experimentation to evaluate the action of a drug or hormone on myometrial electromechanical activities.

Similar to other models, the present model also has some limitations. While it inherits some limitations from the Tong *et al*. model that has been documented elsewhere, the primary limitation of this model is that it has been constructed based on data available in the literature, which were obtained over many years by different groups under variant conditions and from different species. The data used to build this model comes primarily from voltage-clamp experiments on late-pregnant rat cells. In some cases, where rat data was not available human data was substituted in its place; for example the current density of the T-type calcium channel was fitted to human values using data from α1G/Cav3.1 rat genes^45,46^. Ideally in future data obtained from the same species and consistent experimental conditions will be helpful to further improve the model development.

## Electronic supplementary material

Supplementary Information

## References

[1] Beck S (2010). The worldwide incidence of preterm birth: a systematic review of maternal mortality and morbidity. Bull. World Health Organ..

[2] Swamy GK, Østbye T, Skjærven R (2008). Association of Preterm Birth With Long-term Survival, Reproduction, and Next-Generation Preterm Birth. JAMA.

[3] Lammers WJEP, Stephen B, Al-Sultan MA, Subramanya SB, Blanks AM (2015). The location of pacemakers in the uteri of pregnant guinea pigs and rats. Am. J. Physiol. Regul. Integr. Comp. Physiol..

[4] Young RC (2007). Myocytes, myometrium, and uterine contractions. Ann. N. Y. Acad. Sci..

[5] Taggart MJ, Wray S (1998). Contribution of sarcoplasmic reticular calcium to smooth muscle contractile activation: gestational dependence in isolated rat uterus. J. Physiol..

[6] Lutton, E. J., Lammers, W. J. E. P., James, S., Berg, H. A. van den & Blanks, A. M. Identification of uterine pacemaker regions at the myometrial-placental interface. *bioRxiv* 152678 10.1101/152678 (2017). 10.1113/JP275688 PMC6046083 29704394

[7] Burdyga T, Borisova L, Burdyga AT, Wray S (2009). Temporal and spatial variations in spontaneous Ca events and mechanical activity in pregnant rat myometrium. Eur. J. Obstet. Gynecol. Reprod. Biol..

[8] Matthew A, Shmygol A, Wray S (2004). Ca2+ entry, efflux and release in smooth muscle. Biol. Res..

[9] Yoshino M, Wang SY, Kao CY (1997). Sodium and Calcium Inward Currents in Freshly Dissociated Smooth Myocytes of Rat Uterus. J. Gen. Physiol..

[10] Inoue Y, Sperelakis N (1991). Gestational change in Na+ and Ca2+ channel current densities in rat myometrial smooth muscle cells. Am. J. Physiol..

[11] Bursztyn L, Eytan O, Jaffa AJ, Elad D (2007). Mathematical model of excitation-contraction in a uterine smooth muscle cell. Am. J. Physiol. Cell Physiol..

[12] Rihana S, Terrien J, Germain G, Marque C (2009). Mathematical modeling of electrical activity of uterine muscle cells. Med. Biol. Eng. Comput..

[13] Tong W-C (2011). A Computational Model of the Ionic Currents, Ca2+ Dynamics and Action Potentials Underlying Contraction of Isolated Uterine Smooth Muscle. PLoS ONE.

[14] Tong W-C, Tribe RM, Smith R, Taggart MJ (2014). Computational modeling reveals key contributions of KCNQ and hERG currents to the malleability of uterine action potentials underpinning labor. PloS One.

[15] Atia J (2016). Reconstruction of Cell Surface Densities of Ion Pumps, Exchangers, and Channels from mRNA Expression, Conductance Kinetics, Whole-Cell Calcium, and Current-Clamp Voltage Recordings, with an Application to Human Uterine Smooth Muscle Cells. PLoS Comput. Biol..

[16] Fast VG (2005). Simultaneous optical imaging of membrane potential and intracellular calcium. J. Electrocardiol..

[17] Christoph J, Schröder-Schetelig J, Luther S (2017). Electromechanical optical mapping. Prog. Biophys. Mol. Biol..

[18] Choi, C. Y. A biophysically detailed mathematical model of a single late pregnant rat myometrial cell (Manchester eScholar - The University of Manchester). Available at: https://www.escholar.manchester.ac.uk/uk-ac-man-scw:110633 (Accessed: 8th March 2012).

[19] Yang J, Clark JW, Bryan RM, Robertson C (2003). The myogenic response in isolated rat cerebrovascular arteries: smooth muscle cell model. Med. Eng. Phys..

[20] Hai CM, Murphy RA (1988). Cross-bridge phosphorylation and regulation of latch state in smooth muscle. Am. J. Physiol..

[21] Landa J, West TC (1956). Transmembrane potentials and contractility in the pregnant rat uterus. Am. J. Physiol..

[22] Chard, T. *The Uterus*. (Cambridge University Press, 1994).

[23] Bengtsson B, Chow EM, Marshall JM (1984). Activity of circular muscle of rat uterus at different times in pregnancy. Am. J. Physiol..

[24] Wray S (1993). Uterine contraction and physiological mechanisms of modulation. Am. J. Physiol..

[25] Kuriyama H, Suzuki H (1976). Changes in electrical properties of rat myometrium during gestation and following hormonal treatments. J. Physiol..

[26] Xu J (2015). The role of cellular coupling in the spontaneous generation of electrical activity in uterine tissue. PloS One.

[27] Inoue Y, Okabe K, Soeda H (1999). Augmentation and suppression of action potentials by estradiol in the myometrium of pregnant rat. Can. J. Physiol. Pharmacol..

[28] Martin C (1999). Pregnant rat myometrial cells show heterogeneous ryanodine- and caffeine-sensitive calcium stores. Am. J. Physiol. -Cell Physiol..

[29] Shmigol AV, Eisner DA, Wray S (1998). Properties of Voltage-Activated [Ca2+]i Transients in Single Smooth Muscle Cells Isolated from Pregnant Rat Uterus. J. Physiol..

[30] Sanborn BM (2000). Relationship of ion channel activity to control of myometrial calcium. J. Soc. Gynecol. Investig..

[31] Shmigol AV, Eisner DA, Wray S (2001). Simultaneous measurements of changes in sarcoplasmic reticulum and cytosolic [Ca2+] in rat uterine smooth muscle cells. J. Physiol..

[32] Okabe K, Inoue Y, Soeda H (1999). Estradiol inhibits Ca2+ and K+ channels in smooth muscle cells from pregnant rat myometrium. Eur. J. Pharmacol..

[33] Ma Q (2013). Inhibitory effects of 17beta-estradiol on spontaneous and activated contraction of rat uterus smooth muscle. Zhongguo Ying Yong Sheng Li Xue Za Zhi Zhongguo Yingyong Shenglixue Zazhi Chin. J. Appl. Physiol..

[34] Arrowsmith S, Wray S (2014). Oxytocin: its mechanism of action and receptor signalling in the myometrium. J. Neuroendocrinol..

[35] Zafrah, H. A. & Alotaibi, M. F. The effect of extracellular ATP on rat uterine contraction from different gestational stages and its possible mechanisms of action. *J. Basic Clin. Physiol. Pharmacol*. 10.1515/jbcpp-2016-0118 (2017). 10.1515/jbcpp-2016-0118 28358713

[36] Noble K, Zhang J, Wray S (2006). Lipid rafts, the sarcoplasmic reticulum and uterine calcium signalling: an integrated approach. J. Physiol..

[37] Alotaibi M, Arrowsmith S, Wray S (2015). Hypoxia-induced force increase (HIFI) is a novel mechanism underlying the strengthening of labor contractions, produced by hypoxic stresses. Proc. Natl. Acad. Sci. USA.

[38] Hanley J-A, Weeks A, Wray S (2015). Physiological increases in lactate inhibit intracellular calcium transients, acidify myocytes and decrease force in term pregnant rat myometrium. J. Physiol..

[39] Inoue Y, Shimamura K, Sperelakis N (1992). Oxytocin actions on voltage-dependent ionic channels in pregnant rat uterine smooth muscle cells. Can. J. Physiol. Pharmacol..

[40] King JF, Flenady V, Papatsonis D, Dekker G, Carbonne B (2003). Calcium channel blockers for inhibiting preterm labour; a systematic review of the evidence and a protocol for administration of nifedipine. Aust. N. Z. J. Obstet. Gynaecol..

[41] Arrowsmith S, Kendrick A, Hanley J-A, Noble K, Wray S (2014). Myometrial physiology–time totranslate?. Exp. Physiol..

[42] Parkington HC, Tonta MA, Brennecke SP, Coleman HA (1999). Contractile activity, membrane potential, and cytoplasmic calcium in human uterine smooth muscle in the third trimester of pregnancy and during labor. Am. J. Obstet. Gynecol..

[43] Tribe RM (2001). Regulation of human myometrial contractility during pregnancy and labour: are calcium homeostatic pathways important?. Exp. Physiol..

[44] Maigaard S, Forman A, Brogaard-Hansen KP, Andersson KE (1986). Inhibitory effects of nitrendipine on myometrial and vascular smooth muscle in human pregnant uterus and placenta. Acta Pharmacol. Toxicol. (Copenh.).

[45] Wray S, Kupittayanant S, Shmygol A, Smith RD, Burdyga T (2001). The Physiological Basis of Uterine Contractility: A Short Review. Exp. Physiol..

[46] Wu X, Morgan KG, Jones CJ, Tribe RM, Taggart MJ (2008). Myometrial mechanoadaptation during pregnancy: implications for smooth muscle plasticity and remodelling. J. Cell. Mol. Med..

[47] Arrowsmith, S., Quenby, S., Weeks, A., Burdyga, T. & Wray, S. Poor Spontaneous and Oxytocin-Stimulated Contractility in Human Myometrium from Postdates Pregnancies. *PLoS ONE* 7 (2012). 10.1371/journal.pone.0036787 PMC3349652 22590608

[48] Serrano JR, Perez-Reyes E, Jones SW (1999). State-dependent inactivation of the alpha1G T-type calcium channel. J. Gen. Physiol..

[49] Hering J, Feltz A, Lambert RC (2004). Slow Inactivation of the CaV3.1 Isotype of T-Type Calcium Channels. J. Physiol..

