# Supplementary material for: A computational model of excitation and contraction in uterine myocytes from the pregnant rat
Source: Sci Rep. 2018 Jun 14;8:9159. doi: 10.1038/s41598-018-27069-x (PMC6002389; doi:10.1038/s41598-018-27069-x)
Supplement: Supplementary file 1 — Supplementary Information [file 41598_2018_27069_MOESM1_ESM.pdf]

A computational model of excitation and contraction in uterine myocytes from the pregnant rat

Craig P. Testrow[1], Arun V. Holden[2], Anatoly Shmygol[3], Henggui Zhang[1,4,5,6]
[1]The University of Manchester, School of Physics and Astronomy, Manchester, M13 9PL, UK
[2]The University of Leeds, School of Biomedical Sciences, Leeds, LS2 9JT, UK
[3]United Arab Emirates University, College of Medicine and Health Sciences, Department of Physiology, Al Ain, P.O. Box 17666, UAE
[4]School of Computer Science and Technology, Harbin Institute of Technology (HIT), Harbin, 150001, China
[5]Space Institute of Southern China, Shenzhen, 518117, China
[6]Key laboratory of Medical Electrophysiology, Ministry of Education, Collaborative Innovation Center for Prevention and Treatment of Cardiovascular Disease/Institute of Cardiovascular Research, Southwest Medical University, Luzhou, 646000, China

Supplement A – Electromechanical model

This supplement details elements of the ion channels, exchangers and pumps that constitute the electrophysiological element of our single uterine cell model that were not included in the main body of this text.

L-type calcium current - I_{CaL}

I_{CaL} is the major contributor to the total inward current and is present in both rat and human uterine smooth muscle cells. It is the main entry route for calcium, acting as the trigger for myometrial contraction [1]–[4]. The voltage-dependence of the steady-state of the activation and inactivation processes in the Tong et al. model has been altered (Fig A1A and A1B). These changes ensure that the I-V relationship for I_{CaL} (Fig A1F) provide a close fit to the most recent data available (Jones et al 2004) over the physiologically relevant range from -60 to 40 mV. The activation and inactivation curves have been shifted by -7 and + 5mV.

I_{CaL} activates between -40 to -30 mV, peaks between -10 to 10 mV and has a reversal potential E_{CaL} ≈ 45 mV to 60 mV at 30-35°C at an extracellular Ca^{2+} concentration of 1.2-1.5 mM [5], [6]. Currently there is no data specific to myometrial cells, but it has been reported that in other cell types where I_{CaL} is present, it is permeable to other cations [7]. Therefore, the commonly used Goldman-Hodgkin-Katz formulation is not used here; E_{CaL} has instead been fixed at 45 mV, in agreement with experimental data [5], [6].

Different steady-state values for activation and inactivation have been reported, possibly reflecting different $[Ca^{2+}]_o$ employed between studies or differing residual hormonal influences. The half-activation and I-V relationships are right-shifted by ≈ 15 mV when $[Ca^{2+}]_o$ was increased from 3 mM to 30 mM [8]. Yamamoto [6] showed that the I-V relationship was reduced and the half-inactivation left-shifted in myometrial cells exposed to estradiol, which increases near term in rodents. The model uses data sets that are close to the control sets of Yamamoto [6], which are representative of the I_{CaL} steady-state values from a collection of studies [3], [8].

Due to the lack of available data for voltage-dependent activation time constants, they were extracted from published current tracings, specifically from Jones et al. [9], by fitting the initial few tens of milliseconds of raw data; this is undertaken with the assumption that I_{Cl} would be slower than I_{CaL}. The qualification for this assumption is that the activation times for I_{CaL} and I_{Cl} in other smooth muscles are 2-8 ms and > 50 ms respectively. The initial fast inward current was blocked by nifedipine, permeable to Ba^{2+} and increased by L-type calcium channel agonist Bay K8644; thus, it was attributed to I_{CaL}. The fast inactivation is voltage-independent at ≈ 12 ms and the slow inactivation is voltage-dependent with a minimum ≈ 55 ms at V = 0 mV.

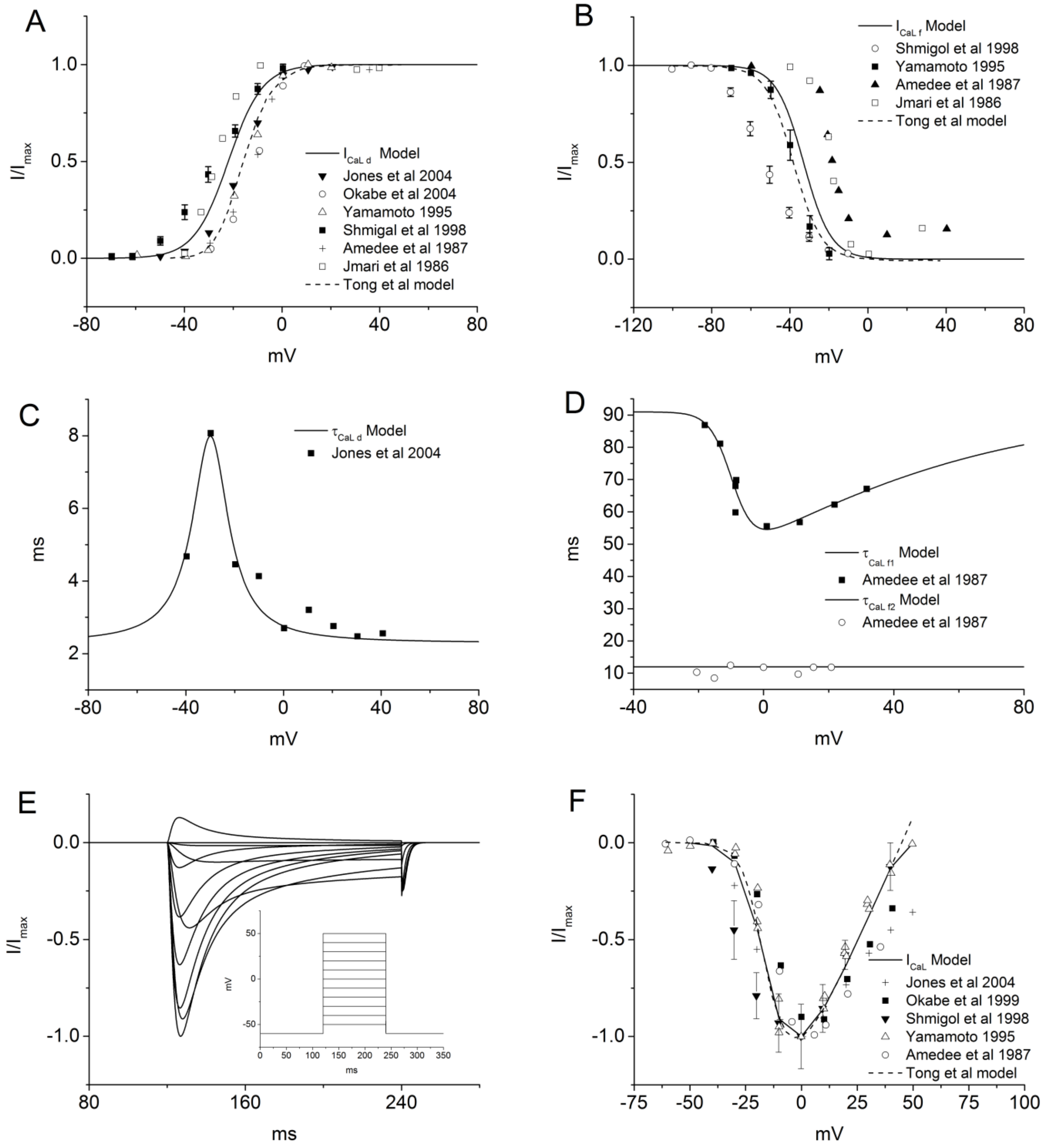

Fig A1. Voltage dependence and dynamics of L-type calcium current model – I_{CaL} A: the activation and **B**: inactivation steady states of I_{CaL}. **C** and **D:** the activation and inactivation time constants of I_{CaL}, respectively.**E:** the normalised current-time traces resulting from a voltage-step protocol of -50 mV to 50 mV in steps of 10 mV with a holding potential of -60 mV, used to establish the I-V relationship of I_{CaL}. **F:** the normalised peak I-V relationship of I_{CaL}.

T-type calcium current – I_{CaT}

There have been reports of I_{CaT} in human myometrial cells [4], [10]. Detailed electrophysiological data of cells expressing rat α1G/Cav3.1 are available [11], [12]. Spontaneous contractions in strips of pregnant rat myometrial tissue were inhibited by the reputed T-type calcium channel blockers mibefradil, NNC 55-0396 (a non-hydrolysable analogue of mibefradil) and Ni^{+} [13], [14]. In addition Ohkubo et al. [15] showed that the expressions of mRNA encoding for the α1G and α1H protein subunits of the T-type calcium channel were gestationally regulated in murine

myometrial cells. As a result, this model has been developed from the electrophysiological characteristics of the rat α1G/Cav3.1clona expression cell data, which was recorded at room temperature [11] and adjusted to the human myometrial cell current density [4], [10]. The activation and inactivation steady-state values and the I-V relationships of these different datasets are similar.

I_{CaT} activates at V ≈ -60 mV, peaks between -20 to -30 mV and has fast activation, but inactivation varying between 7 to 100 ms [4], [10]–[12]. This might be due to different external divalent cation concentrations used between experiments.

Calcium pump current - I_{Capump}

The calcium pump (plasma membrane Ca2+ ATP-ase) is an ionic transporter that maintains and regulates the calcium concentrations between the cytoplasm, sarcoplasmic and endoplasmic reticulum (SR and ER) in the cells [16]–[18]. There is a disparity between the concentrations of calcium ions in the cytosol (0.1 μM) and the ER and SR (0.1 to 1 mM) [19]; thus Ca^{2+} ions must be pumped against the concentration gradient, using energy obtained from the ATP chemical energy store. We assume an instantaneous pump with quick bindings resulting in rapid equilibrium using an empirical Hill-type formula.

Sodium/potassium exchanger current – I_{NaK}

This exchanger current plays a major role in regulating intra and extracellular sodium and potassium cations and has been found in late pregnant rat [20], [21] and human [1] myometrial cells. A net negative charge within the cell is achieved by using energy derived from hydrolysed ATP to expel three Na^+ ions for each two K^+ ions brought in. I_{NaK} reportedly increases during pregnancy in the rat myometrium [21].

This model adopts and modifies the formulae from the Luo-Rudy mammalian ventricular model for I_{NaK}, due to the absence of information relating to its role in the myometrium [22]. The inhibition of the exchanger changes dynamically, affecting the activities of K+ and its dependent currents, leading to a bursting AP.

Fast sodium current – I_{Na}

I_{Na} has been found in pregnant human myometrial cells [23] and a fraction of late pregnant rat myometrial cells [4]. This channel is sensitive to tetrotodoxin and sagitoxin. It may be responsible for the fast upstroke of AP generation, the formation of the gap junction and quick excitation propagation during parturition [24]. Studies have found that the number density of sodium channels in the rat increases towards term [25].

I_{Na} activates at V ≈ -50 mV, reaches its peak between -10 to 10 mV within ≈ 1 ms and is almost completely inactivated after 10 to 20 ms [3], [8].

Sodium/calcium exchanger current – I_{NaCa}

I_{NaCa} expels a Ca^{2+} ion and brings in three Na^+ ions against the concentration gradient. Abe [26] found that the pre-potential and spontaneous spike discharge disappears at low $[Na^+]_o$, but the tissue remains excitable; a depolarising current stimulus causes larger, faster spikes. This confirms the existence of the electrogenic I_{NaCa} in the rat myometrium. We also borrow from cardiac models for this current, particularly the Hund-Rudy canine ventricular model [27], to make up for the lack of available data.

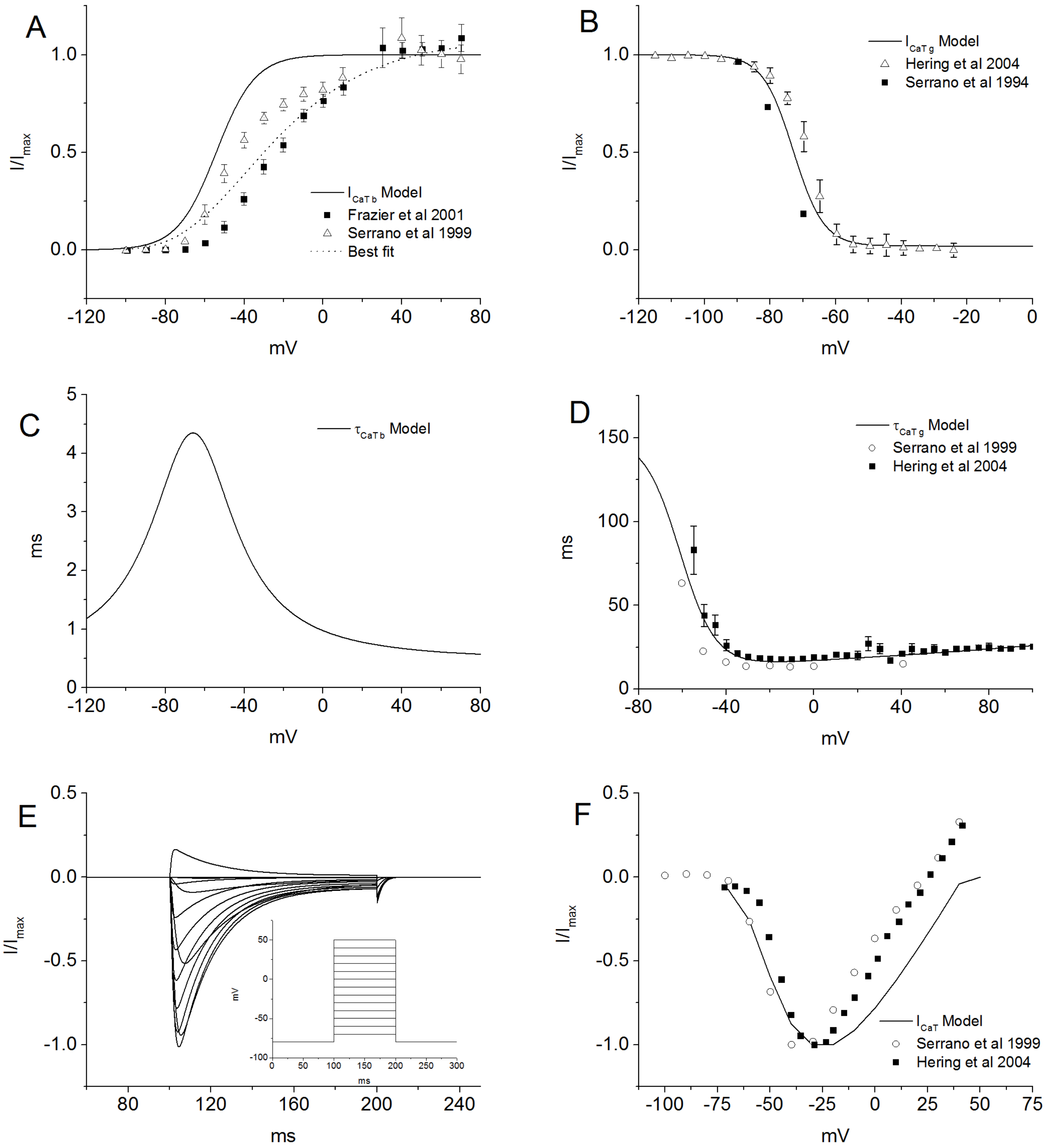

Fig A2. T-type calcium current – I_{CaT} A and B: the activation and inactivation steady states of I_{CaT}, respectively. This is not an ideal fit to the steady state data, but produced a closer reproduction of the I_{CaT} I-V relationship. **C** and **D**: the activation and inactivation time constants of I_{CaT}, respectively. **E:** the normalised current-time traces resulting from a voltage-step protocol of -70 mV to 50 mV in steps of 10 mV with a holding potential of -80 mV, used to establish the I-V relationship of I_{CaT}. **F:** the normalised peak I-V relationship of I_{CaT}.

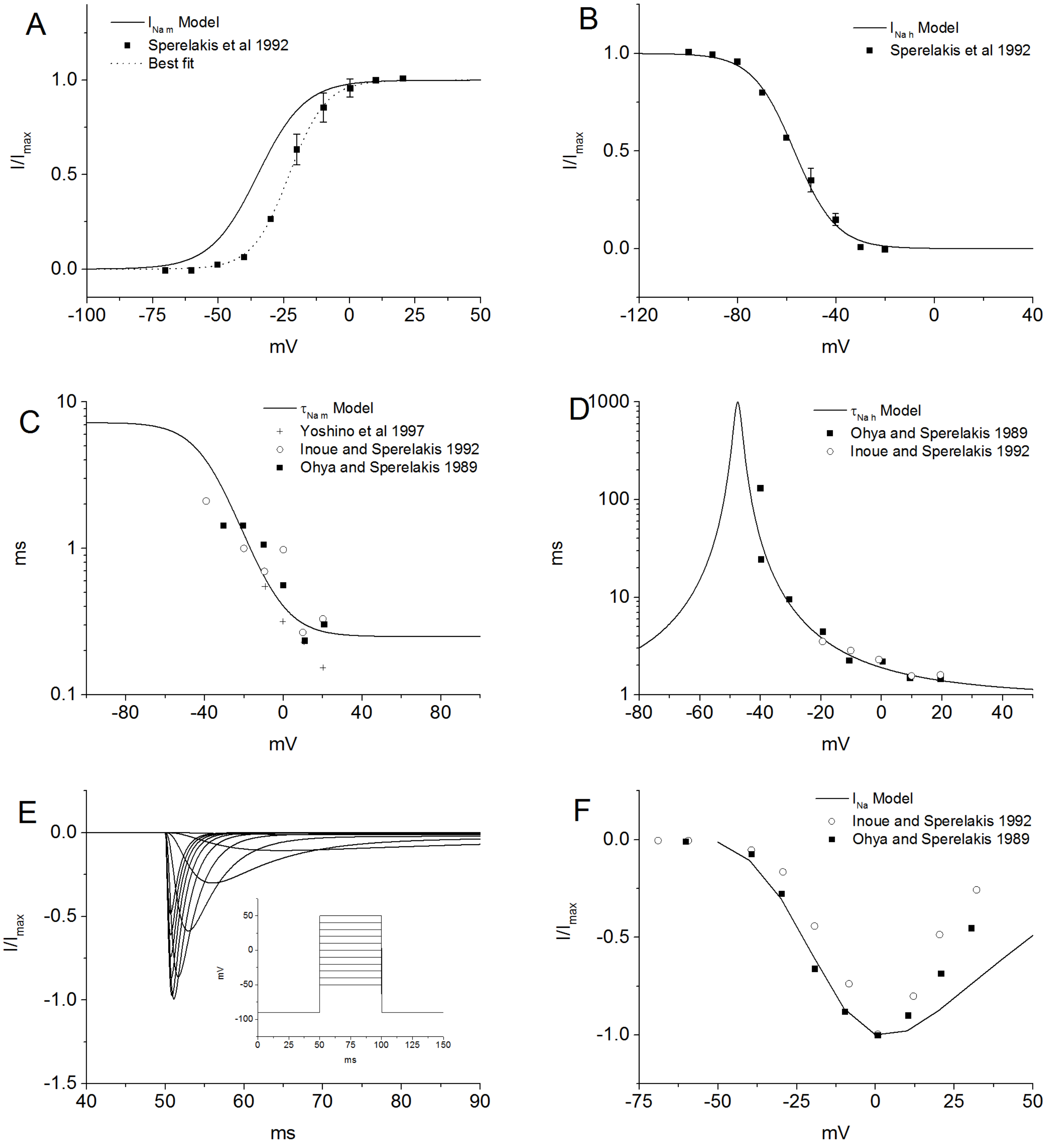

Fig A3. Fast sodium current – I_{Na} A and **B**: the activation and inactivation steady states of I_{Na}, respectively. **C** and **D**: the activation and inactivation time constants of I_{Na}, respectively. **E:** the normalised current-time traces resulting from a voltage-step protocol of -50 mV to 50 mV in steps of 10 mV with a holding potential of -90 mV, used to establish the I-V relationship of I_{Na}. **F:** the normalised peak I-V relationship of I_{Na}.

Voltage-dependent potassium currents – I_{K1} and I_{K2}

I_{K1} and I_{K2} play a role in repolarising the cell. We have altered the activation steady state relationship of I_{K2} from the Tong et al. model in order to provide a better fit to the I-V relationship obtained by Knock and Aaronson (Fig A5A).

Knock et al. [28] separated I_{K1} and I_{K2} myometrial potassium currents and categorized them by their inactivation properties and sensitivity to pharmacological blockers of varying channel subtype specificity [28], [29]. I_{K1} and I_{K2} have rectifying properties and were found in myometrial cells of late pregnant rats [29] and humans [28]; they have very slow dynamics in comparison to other membrane currents and are unevenly distributed between cells. Their primary role appears to be to reset the action potential to its resting state by repolarising the cell membrane.

I_{K1} and I_{K2} have similar voltage-dependent kinetics. I_{K1} activates between V ≈ -50 to -40 mV and half-inactivates between -62 to -68 mV. I_{K2} activates between V ≈ -40 to -30 mV and half-inactivates between -30 to -20 mV. I_{K2} inactivates faster than I_{K1}. Our model took advantage of the more abundant information on the electrophysiological characteristics of human myometrial I_{K1} and I_{K2}, complementing them with data from rat myometrium. I_{K1} and I_{K2} were separated by their activation thresholds, inactivation properties and current sensitivities to 4-aminopyridine (4-AP) and TEA. The Tong et al fit of the IK2 activation steady state was adjusted to allow a better fit to the unpublished I-V curve data from Knock and Aaronson.

A-type transient potassium current – I_{KA}

I_{KA} has very fast activation and inactivation kinetics, is found in both rat and human myometrial cells (though it is rare in pregnant cells), is sensitive to 4-AP and insensitive to TEA [30]. It activates at V ≈ -40 mV, peaks within ≈ 10 ms and is almost completely inactivated within 50 ms [2], [30]. In human myometrium it has a half-inactivation ≈ -70 mV and a slope factor ≈ 5 mV [28], [30]. These are similar characteristics to the transient potassium current in myometrial cells isolated from immature rats.

Calcium activated potassium current - $I_{K(Ca)}$

$I_{K(Ca)}$ is associated with setting the resting membrane potential and regulating the shape and duration of the action potential. It may be responsible for up to 10% of the total K^+ current in late pregnant rate myometrial cells [29]. Quantitative data for the dynamics of this channel in the myometrium is lacking and the equations developed for rat mesenteric smooth muscle have been used [31]. Calcium activated potassium currents provide a means of burst termination in pancreatic cells (Chay Keizer model) and neurones (Chay Fan model).

Hyperpolarisation-activated current – I_h

This current has reportedly been found in the circular and longitudinal cells of the pregnant murine myometrium [32], [33] and is otherwise known as the funny current (I_f) in cardiac cells. It is activated by hyper polarisation of the membrane potential, below -60 mV, has a half-maximal activation around -85 mV and does not inactivate. In voltage clamp experiments the activation is slow, taking > 1 s. K^+ ions permeate this channel more readily than Na^+ ions, with a ratio of P_{Na}/P_K = 0.35. The reversal potential E_h ≈ -20 mV is dependent on the extracurricular concentration of K^+ and Na^+; and excess of K^+ increases the current amplitude and a low measure of Na^+ decreases it, while Cs^+ blocks the channel. This current is activated at the resting membrane potential and its role is to maintain this potential and spontaneous activity within the myometrium.

Data for this channel has been found experimentally by Okabe et al. [32] and Satoh [33]. Temperatures ranged from 24-30°C; the time constant τ_h was adjusted to compensate, with a Q_{10} value of 3.5. The single cell model was developed for a longitudinal cell. The circular cell half-activation has been shifted by -15 mV to match the longitudinal data. At room temperature, with a holding potential of -50 mV, I_h has a current density of -1.03 $pApF^{-1}$.

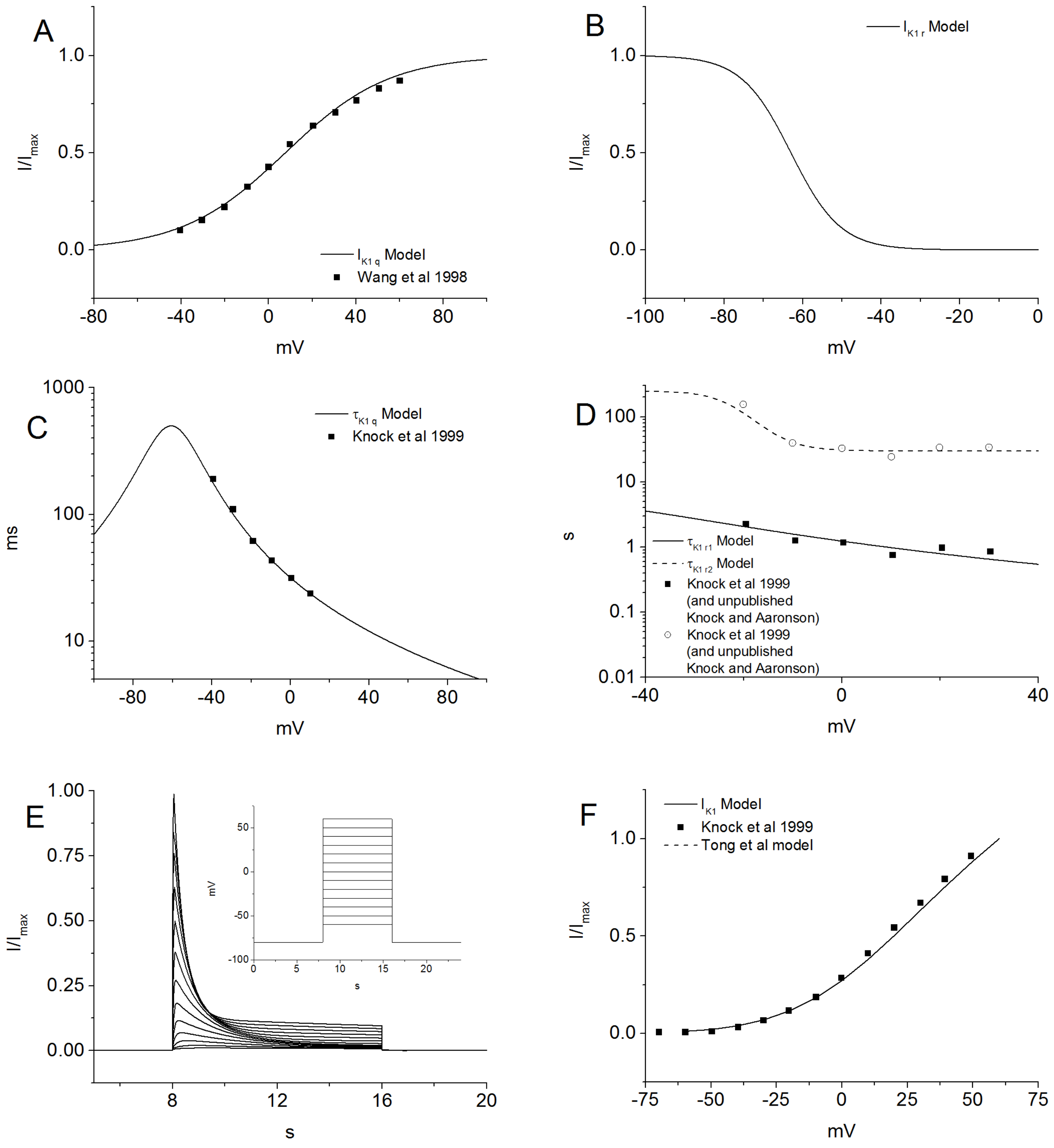

Fig A4. Voltage-dependent potassium current – I_{K1} A and **B**: the activation and inactivation steady states of I_{K1}, respectively. **C** and **D**: the activation and inactivation time constants of I_{K1}, respectively. **E:** The normalised current-time traces resulting from a voltage-step protocol of -60 mV to 60 mV in steps of 10 mV with a holding potential of -80 mV, used to establish the I-V relationship of I_{K1}. **F**: the normalised peak I-V relationship of I_{K1}.

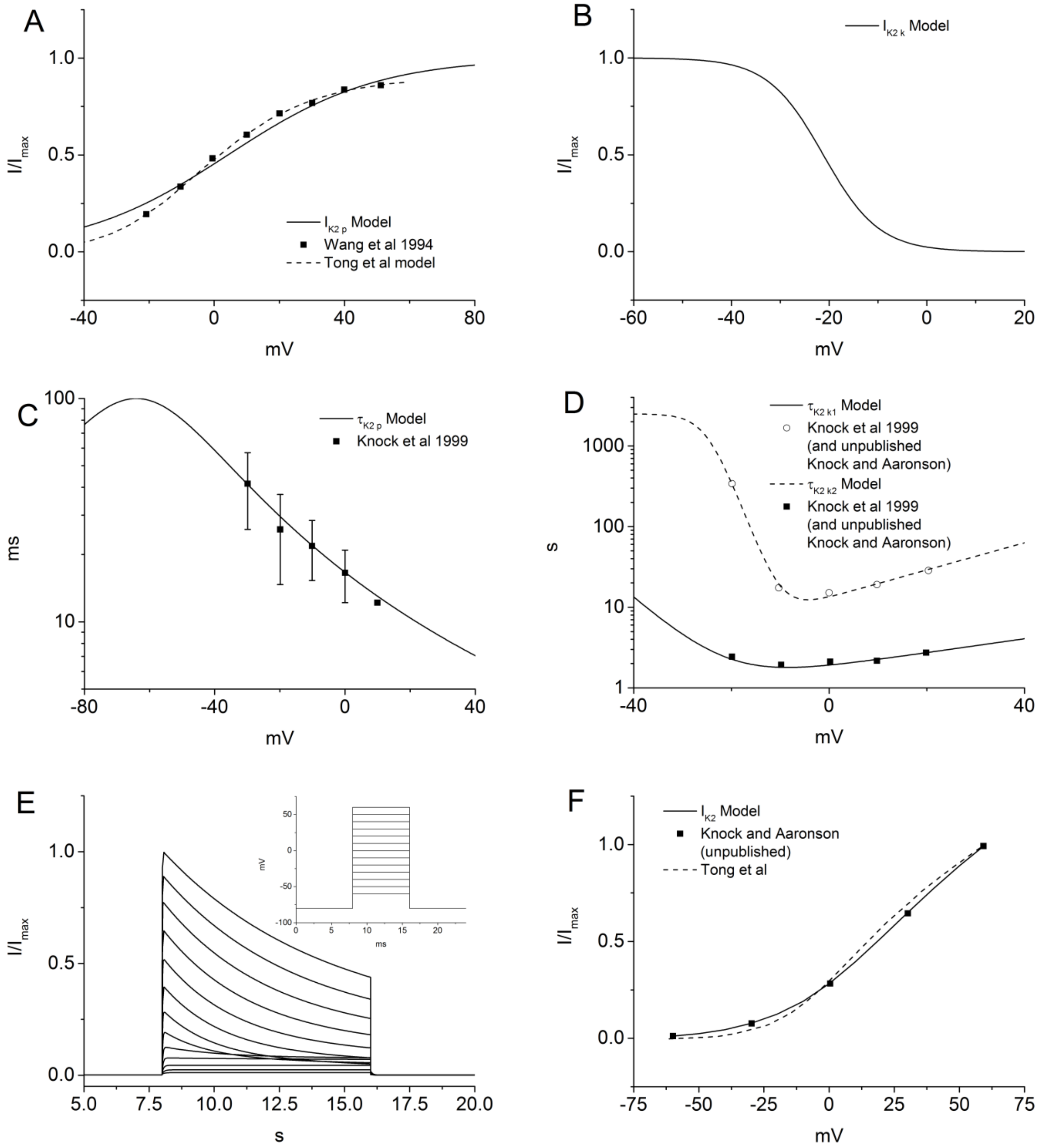

Fig A5. Voltage-dependent potassium current – I_{K2} A and **B:** the activation and inactivation steady states of I_{K2}, respectively. **C** and **D:** the activation and inactivation time constants of I_{K2}, respectively. **E:** The normalised current-time traces resulting from a voltage-step protocol of -60 mV to 60 mV in steps of 10 mV with a holding potential of -80 mV, used to establish the I-V relationship of I_{K2}. **F**: the normalised peak I-V relationship of I_{K2}.

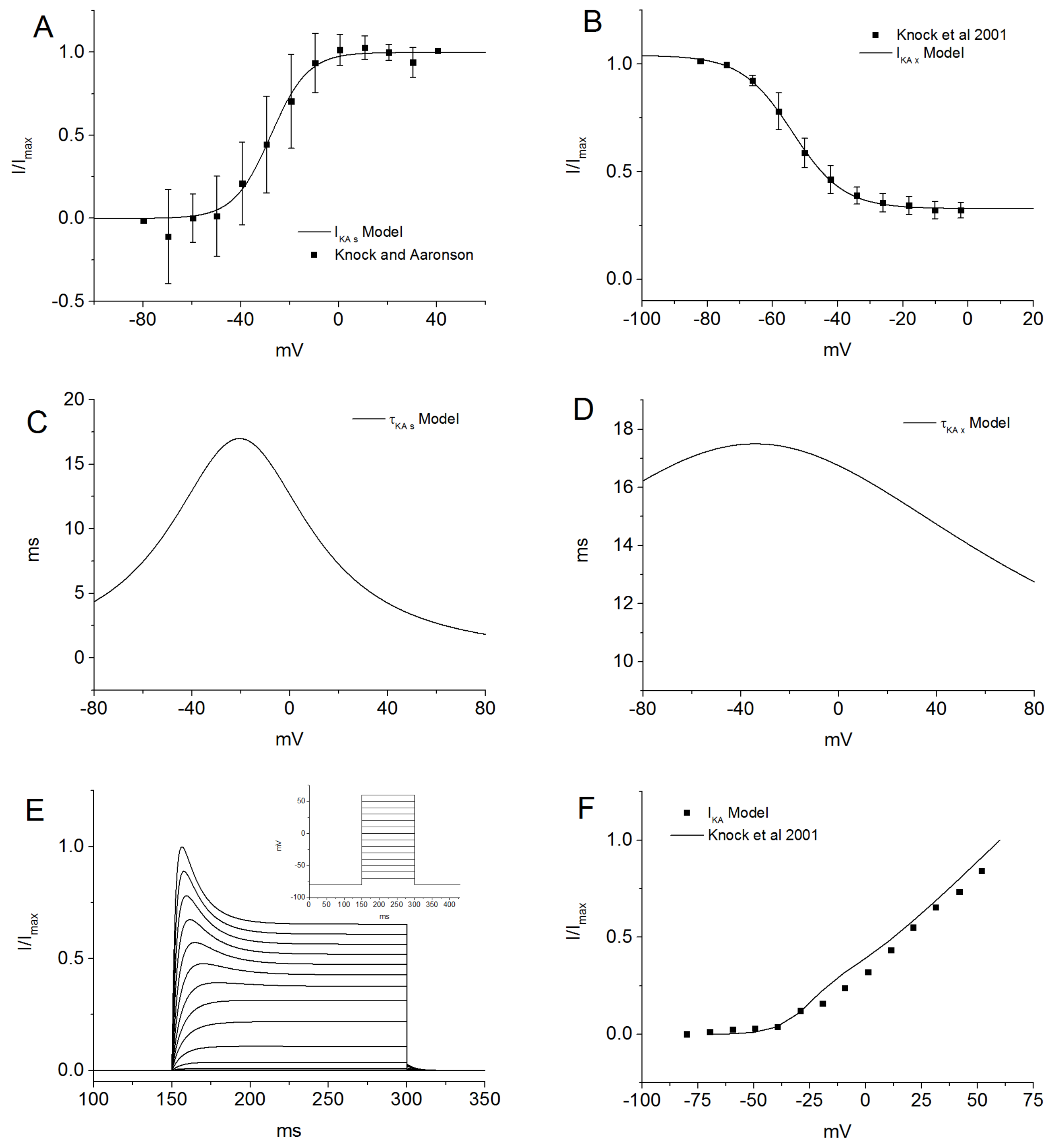

Fig A6. A-type transient potassium current – I_{KA} A and **B**: the activation and inactivation steady states of I_{KA}, respectively. **C** and **D**: the activation and inactivation time constants of I_{KA}, respectively. **E** and **F**: the normalised current-time traces and current-voltage relationship of I_{KA}, respectively. **E:** the normalised current-time traces resulting from a voltage-step protocol of -80 mV to 60 mV in steps of 10 mV with a holding potential of -80 mV, used to establish the I-V relationship of I_{KA}. **D**: the normalised peak I-V relationship of I_{KA}.

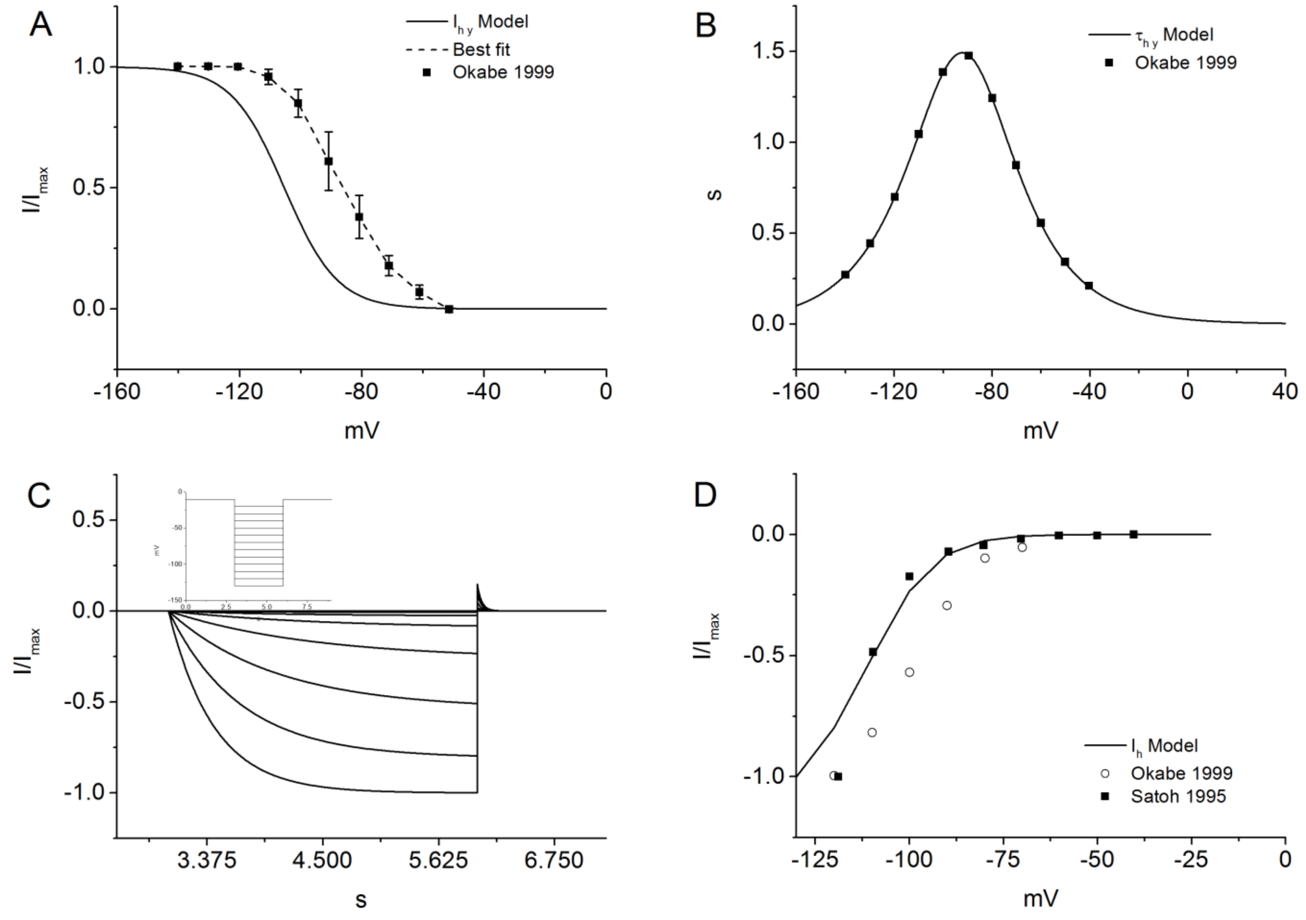

Fig A7. Hyperpolarisation-activated current – I_h **A**: the activation steady state of I_h. The experimental data was taken at temperatures of 24 °C and 30 °C; to compensate a Q_{10} factor of 3.5 was introduced, negatively shifting the activation curve. **B**: the activation time constant of I_h. **C**: the normalised current-time traces resulting from a voltage-step protocol of -20 mV to -130 mV in steps of 10 mV with a holding potential of -10 mV, used to establish the I-V relationship of I_h. **D**: the normalised peak I-V relationship of I_h.

Calcium-activated chloride current – I_{Cl}

Spontaneous transient inward currents (STICs) are pro-contractile currents that exhibit the characteristics of calcium-activated channels (CaCCs) and have been observed in murine and human uterine smooth muscle cells, and in putative pacemaking interstitial cells of Cajal in the intestine [34]. They are blocked by Cl- inhibitors such as niflumic acid and flufenamic acid and are greatly diminished in calcium-free buffers, consistent with their being calcium-activated channels (CaCCs)], showing calcium activation, voltage dependence and a reversal potential of -15 mV [35], [36]. The anoctamin 1 and 2 (ANO1, ANO2) channels are responsible for these currents [36], and their kinetics were formulated from experimental data in Jones et al 2004 [9].

It is believed that CaCCs prolong the duration of contraction in pregnant tissue [9], [37]. They may play a role in depolarising the membrane, leading to increased Ca entry [38], [39]. In the interstitial cells of Cajal (ICC) of the intestines they play a role in pace making; ICC-like cells have been described in the myometrium [40] and Bernstein et al suggest there may be evidence of this role in the myometrium [36]. The contribution of I_{Cl} to the total inward current is a matter for debate, with contrasting reports indicating it is responsible for up to 30% [9] or unimportant [41].

Jones et al [9] assessed the electrophysiology of I_{Cl} in the single cell using two distinct voltage clamp experiments. The activation time relationship of I_{Cl} is relatively complex. In order to provide a more representative activation model for I_{Cl}, its time coefficient relationship has been split into two functions (Fig A8C); the current is calculated differently for voltages above and below 0 mV. The Tong model used an alternative continuous function for the activation time constant and fitted I-V data from Jones 2004. I-V curves for I_{Cl} for our model reproduce those seen in the more recent Bernstein 2014 [36], [42]–[44].

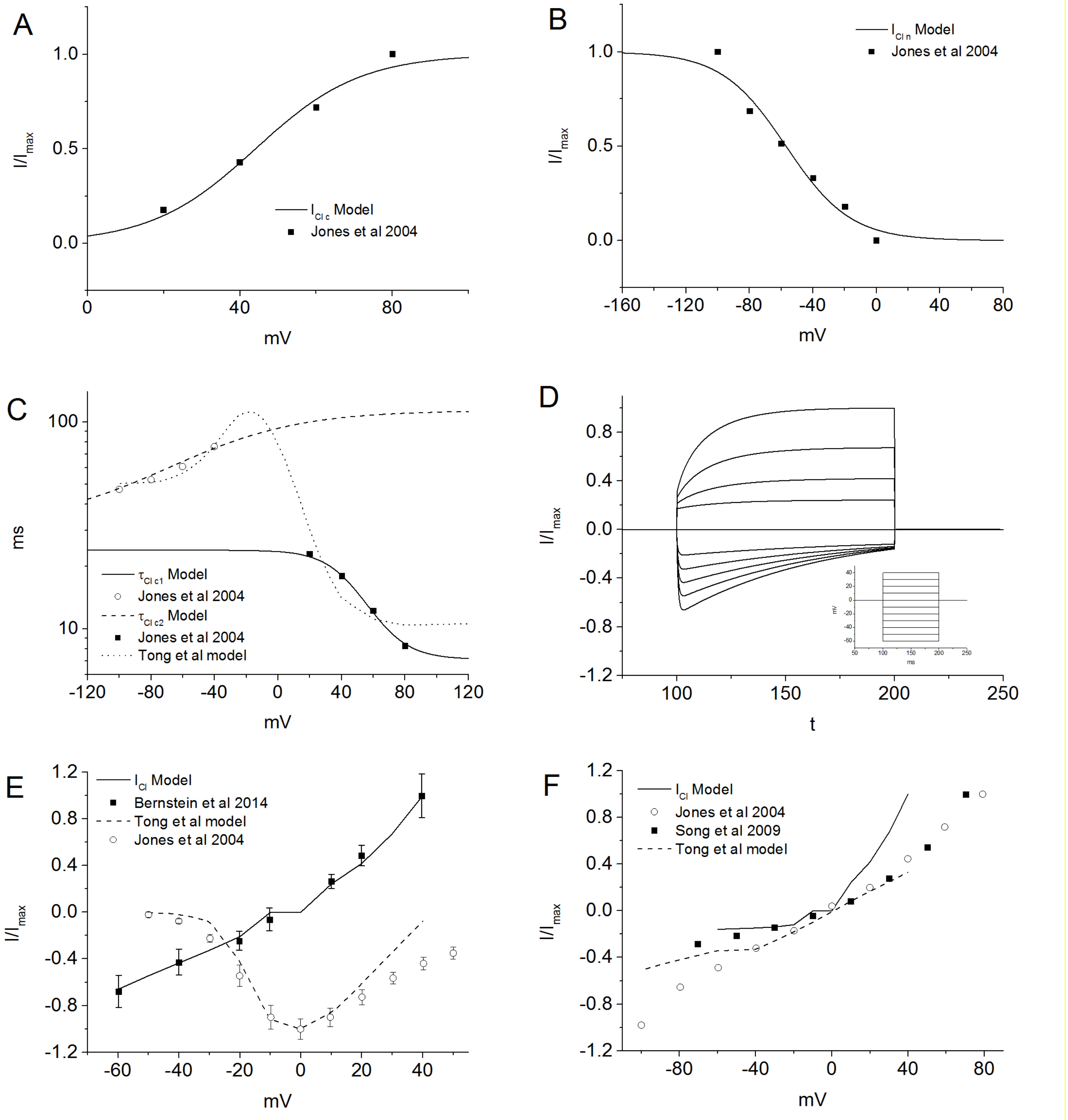

Fig A8. Modifications to the calcium-activated chloride channel – I_{Cl} A and **B:** the activation and inactivation steady states of I_{Cl}, respectively. This updated model includes the inactivation properties. **C:** the activation time constant of I_{Cl} was split into two components. The inactivation time constant was fixed at 0.69 ms (not shown here) **D:** the normalised current-time traces resulting from a voltage-step protocol of -60 mV to 40 mV in steps of 10 mV with a holding potential of 0 mV, used to establish the I-V relationship of I_{Cl}. **E** and **F:** the peak and tail I-V relationships of I_{Cl} respectively. This channel was reformulated to account for new data describing the peak I-V values [36].

Ionic concentration dynamics

$[Ca^{2+}]_i$ increases as the calcium channels respond to a depolarising cell membrane. Na^+, K^+ and Cl^- ion regulation are also regulated by the intracellular ion handling component. Additional Ca^{2+} is also released by the sarcoplasmic reticulum store (SR), by the process of calcium-induced calcium-release (CICR).The $[Ca^{2+}]_i$ –dependent kinetics

respond to this influx of Ca^{2+} into the cytoplasm, leading to the development of contractile forces. Calsequestrin (CSQN) binds to Ca^{2+} in the SR, storing it despite the higher concentration within the SR relative to the cytosol.

The Tong et al. model calculates the calcium current using a simple formula consisting of three components: contributions from the L-type calcium current, the T-type calcium current and the sodium-calcium exchanger. Notably absent from this description are contributions from the non-selective cation current (NSCC), the store-operated non-selective cation current, the calcium pump and the SR currents. We included these factors for a more complete description of the calcium dynamics (B17.1).

Another advance from the previous Tong et al. model was the inclusion of formulae describing the sodium, potassium and chloride ion currents. Changes in the concentration of these ions contribute less significantly to contraction than calcium ions; however their effects are not negligible and should be accounted for in a comprehensive model. We modelled the changes to intracellular ionic concentrations of Ca^{2+}, Na^+, K^- and Cl^- with a series of first order differential equations (B17.2, B17.4, B17.6, and B17.8, respectively). A calcium buffer was implemented to reduce sensitivity to $[Ca^{2+}]_i$ (B17.2).

Changes to intracellular ionic concentrations of Ca^{2+}, Na^+, K^- and Cl^- are described by a series of first order differential equations (B17.2, B17.4, B17.6, and B17.8, respectively). A calcium buffer was implemented to reduce sensitivity to $[Ca^{2+}]_i$ (B17.2).

The calcium currents include contributions from the L-type calcium current, the T-type calcium current, the sodium-calcium exchanger, the non-selective cation current, the store-operated non-selective cation current, the calcium pump and the SR currents in (B17.1). Changes in the concentration of Na^+, K^- and Cl^- do not directly contribute to contraction, but they do influence the behaviour of several ionic currents: the fast sodium current, the voltage-dependent potassium currents, the A-type transient potassium current, the calcium-activated potassium current, the background potassium leakage current, the hyperpolarisation-activated current, the sodium/potassium exchanger current, the sodium/calcium exchanger current, the sodium-potassium-chloride co-transport current, the store-operated non-selective cation current and the non-selective cation current. The feedback from changes to these currents can in turn affect the morphology of the AP, calcium transients and ultimately contractile force. The intracellular ionic concentration dynamics are measureable in in vitro experiments and provide additional constraints on the behaviour of the cell model [45].

Cell contraction

In order to model the contractile activity in the cell we must couple the electrical systems to mechanical systems (Fig 1), using the intracellular calcium concentration as a mediator.

Calcium plays a key role in the mechanical contraction and relaxation of myometrial tissue. Ca^{2+} enters the cell through voltage-gated channels, primarily through the L-type Calcium channel. It can also be released from its store in the sarcoplasmic reticulum (Fig A9) via IP_3 and the ryanodine receptors. Some experimental evidence suggests the SR makes a minimal contribution [46]–[48] to contractile activity; though other findings suggest it may be more significant [49], [50]. Optical measures of intracellular $[Ca^{2+}]$ in tissue preparations have been used as a surrogate measure of myometrial cell excitation [51].

The sarcoplasmic reticulum (SR)

The SR is a calcium store within the cell. Noble et al state that the myometrial SR contributes to sustained depolarisation, bursts of APs and phasic contractions [50]. They remark that the model of the SR developed in cardiac muscle, whereby a small amount of L-type Ca^{2+} entry triggers a substantial rise of cytoplasmic Ca^{2+} via RyR-mediated SR Ca^{2+} release leading to contraction is not substantiated by their findings. Rather the Ca^{2+} released from the SR stimulates store-operated calcium entry (SOCE), meaning that although the SR plays an important role, the greater contribution to contraction in myometrial cells comes from the membrane channels, unlike cardiac myocytes.

We modelled the SR as a two-compartment system; an uptake compartment and a release compartment, as described for vascular smooth muscle cells by Yang et al [52]. Calcium enters the SR via the SERCA pump (I_{up}); the release mechanism is governed by a Hai and Murphy four-state ryanodine receptor model (Fig A10) via the process of CICR (I_{rel}) [53]. The three currents I_{up}, I_{tr} and I_{rel} (B18.1 – B18.3) describe the uptake into the SR, transfer between compartments and release of calcium ions into the cytoplasm.

The Michaelis constant of 1μM suggested by Kapela et al [31] was used rather than the 80 μM used by Yang et al, as it is closer to physiological values. To avoid excessive loading of the SR a leakage component was included, R_{leak}.

This and the release time constant τ_{rel} were adjusted from the Kapela et al model to better fit myometrial $[Ca^{2+}]_i$ data. F_{rel} is a scaling factor used to adjust the magnitude of the release current.

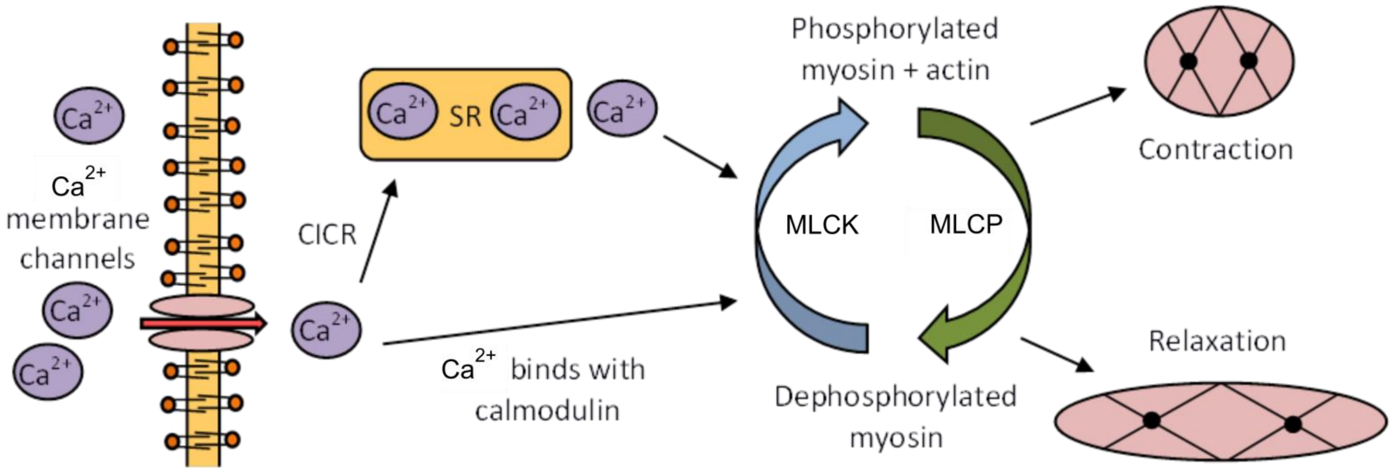

Fig A9. Cell contraction mechanisms in the uterine myocyte [54]**.** Calcium enters the cell through the membrane channels. This begins the process of Calcium Induced Calcium Release (CICR) from the SR. The Ca^{2+} then bonds with calmodulin, activating the Myosin Light-Chain Kinase (MLCK). Phosphorylated myosin can then form cross-bridges with actin, causing the cell to contract. Myosin Light-Chain Phosptase (MLCP) dephosphorylates the myosin leading to relaxation.

The ryanodine receptors (RyR)

In the process of CICR $[Ca^{2+}]_i$ binds to the RyR; they open, allowing calcium from inside the SR store to enter the intracellular space. In order to simulate CICR we have incorporated the model described by Yang et al [52]; the states of the ryanodine receptors are R_{00} (free receptors), R_{10} (activation sites with bound calcium), R_{01} (inactivation sites with bound calcium) and R_{11} (activation and inactivation sites with bound calcium) [52]. Transitions between these states were controlled by the activation and inactivation rate constants, $K_{\pm r1}$ and $K_{\pm r2}$.

The kinetic cross-bridge cycle (KCBC)

We modelled the kinetic cross-bridge cycle using the four-state hypothesis described by Hai and Murphy [53] (Fig A10). Unlike striated and cardiac muscles, smooth muscles do not contain troponin for the calcium to bind to; instead $[Ca^{2+}]_i$ binds with calmodulin and activates the myosin light-chain kinase (MLCK), forming cross-bridges between actin and myosin filaments, via the attached phosphorylated and attached de-phosphorylated non-cycling cross-bridges (AMp and AM) [55], [56], leading ultimately to muscle contraction. There are also free cross-bridges and phosphorylated cross-bridges (M and Mp). The flow between states was determined by the rate constants, $K_{1\text{-}6}$, which are $[Ca^{2+}]_I$ dependent. K_7 was the slow detachment rate constant, which leads to relaxation.

Hai and Murphy established the values for the rate constants by first fitting phosphorylation kinetic data for K_1, K_2, K_5 and K_6. These were then held constant while K_3, K_4 and K_7 were fitted to stress data. The rate-limiting step was revealed to be K_7, the latch-bridge detachment rate.

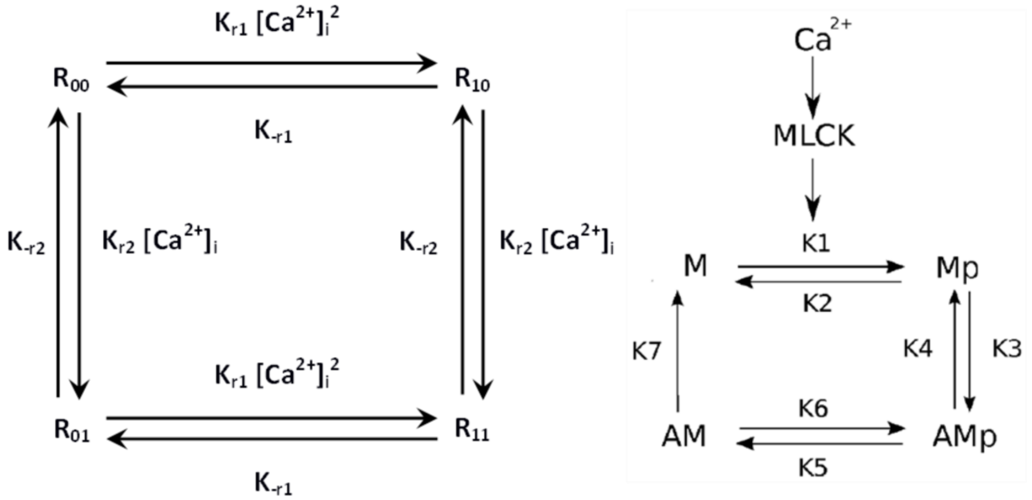

Fig A10. Ryanadine receptor and kinetic cross-bridge cycling models in smooth muscle. Left: Four-state kinetic model of ryanodine receptors from Hai and Murphy [53] and Yang et al [52]. The states are defined as being bound or unbound to Ca^{2+} and if bound, whether the calcium is attached to activation sites, inactivation sites or both. The rate constants K_{r1} and K_{r2} represent the speed that Ca^{2+} binds to the activation and inactivation sites, respectively. Unbinding rates are indicated with a minus sign. **Right:** Four-state kinetic cross-bridge cycling model in smooth muscle adapted from Yang et al [52]. the four-state kinetic cross-bridge cycling model in smooth muscle adapted from Yang et al [52]**.** The states are differentiated by their status as attached or detached, and phosphorylated or dephosphorylated. Cycling between the states is described by the various rate constants, K.

Force production

We modelled force production in the cell as described by Yang et al (Fig A11) [52], using a modification of the three-component Hill model. The cross-bridges in smooth muscles are arranged in parallel with equal spacing; hence force production by the muscles is proportional to the length of the myosin fibre. The total myometrial force is comprised of four components; they are the passive force F_p, cross-bridge elastic force F_x, active force F_a and series visco-elastic force F_s.

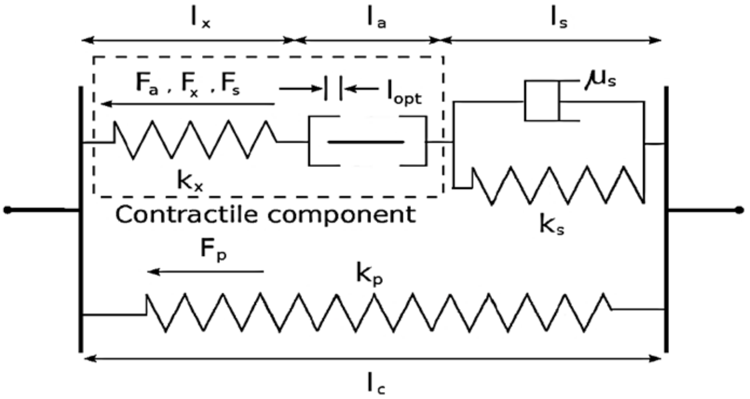

Fig A11. The mechanical model of a smooth muscle cell from Yang et al [52]**.** The four force components F_p, F_a, F_x and F_s couple together to generate tension and stress in the cell. The k values are spring constants and μ_s is the coefficient of viscoelasticity of the dashpot, tasked with absorbing shock from impacts caused by the spring.

F_p (B21.5) is dependent on the length of the cell l_c, taken to be 123 μm. The myometrial cell length for the late pregnant rat can range from 80 to 700 μm [3], [25], [57], [58]. Other parameters necessary to calculate F_p that were not available experimentally for the uterine cell have been substituted for values found in the Yang et al arterial smooth muscle model.

F_x (B21.6) is dependent on the length of the cross-bridge l_x and the length of the active force component l_a. l_{opt} represents the optimal muscle length, i.e. when the maximum force is achieved from the overlapping of the myosin and actin filaments. F_x and F_a coupled to describe the sliding of the myosin and actin filament.

F_a (B21.7) is the component of the force generated by the activity of the cross-bridges. It is characterised by the friction coefficients for the attached phosphorylated (AMp) and attached dephosphorylated (AM) cross-bridges.

F_s (B21.8) is described by a spring and dashpot working in parallel. The force produced by the spring can deform the element and so the dashpot is included as a shock absorber to compensate for the effect. The displacement for the two components is the same and the total force produced by this element is a combination of the elastic and viscous forces. The total force produced by the cell is the sum of F_a and F_p (1.5).

Supplement B – Formulations and parameters

This supplement contains all of the equations and parameters used in our single cell model. Formulae that are not present in Tong et al or have been altered are highlighted in red.

L-type calcium current – I_{CaL}

$$I_{CaL} = \bar{g}_{CaL} d^2 f_{Ca} (0.8 f_1 + 0.2 f_2)(V - E_{CaL})$$ B1.1

$$f_{Ca} = \frac{1}{1 + \frac{[Ca^{2+}]_i}{K_{m,CaL}}}$$ B1.2

$$d_\infty = \frac{1}{1 + e^{\frac{-V-22}{7}}}$$ B1.3

$$f_\infty = \frac{1}{1 + e^{\frac{V+33}{7}}}$$ B1.4

$$\tau_d = 2.29 + \frac{5.7}{1 + \left(\frac{V+29.97}{9}\right)^2}$$ B1.5

$$\tau_{f_1} = 12\ ms$$ B1.6

$$\tau_{f_2} = 90.97 - \frac{90.97}{\left(1 + e^{\frac{V+13.96}{45.38}}\right)\left(1 + e^{\frac{-V-9.5}{3.39}}\right)}$$ B1.7

$$\frac{dd}{dt} = \frac{d_\infty - d}{\tau_d}$$ B1.8

$$\frac{df_1}{dt} = \frac{f_\infty - f_1}{\tau_{f_1}}$$ B1.9

$$\frac{df_2}{dt} = \frac{f_\infty - f_2}{\tau_{f_2}}$$ B1.10

Parameter	Value	Definition
$\bar{g}_{CaL}$	0.318 nS pF^{-1}	Maximum conductance of I_{CaL}
E_{CaL}	45 mV	Reversal potential of I_{CaL}
$K_{m,CaL}$	0.6 μM	Half saturation concentration of I_{CaL}

Table B1. L-type calcium current parameters

T-type calcium current – I_{CaT}

$$I_{CaT} = \bar{g}_{CaT} b^2 g (V - E_{CaT})$$ B2.1

$$b_\infty = \frac{1}{1 + e^{\frac{-V-43}{6}}}$$ B2.2

$$g_\infty = 0.02 + \frac{0.98}{1 + e^{\frac{V+72.98}{4.64}}}$$ B2.3

$$\tau_b = 0.45 + \frac{3.9}{1 + \left(\frac{V+66}{26}\right)^2}$$ B2.5

$$\tau_g = 150 - \frac{150}{\left(1+e^{\frac{V-417.43}{203.18}}\right)\left(1+e^{\frac{-V+61.11}{8.07}}\right)}$$ B2.5

$$\frac{db}{dt} = \frac{b_\infty - b}{\tau_b}$$ B2.6

$$\frac{dg}{dt} = \frac{g_\infty - g}{\tau_g}$$ B2.7

Parameter	Value	Definition
$\bar{g}_{CaT}$	0.02319 nS pF^{-1}	Maximum conductance of I_{CaT}
E_{CaT}	42 mV	Reversal potential of I_{CaT}

Table B2. T-type calcium current parameters

Fast sodium current – I_{Na}

$$I_{Na} = \bar{g}_{Na} m^3 h (V - E_{Na})$$ B3.1

$$E_{Na} = \frac{RT}{F} ln\left(\frac{[Na]_o}{[Na]_i}\right)$$ B3.2

$$m_\infty = \frac{1}{1+e^{\frac{-V+35}{9}}}$$ B3.3

$$h_\infty = \frac{1}{1+e^{\frac{V+57}{8}}}$$ B3.4

$$\tau_m = 0.25 + \frac{7}{1+\left(\frac{V+38}{10}\right)}$$ B3.5

$$\tau_h = 0.9 + \frac{1002.85}{1+\left(\frac{V+47.5}{1.5}\right)^2}$$ B3.6

$$\frac{dm}{dt} = \frac{m_\infty - m}{\tau_m}$$ B3.7

$$\frac{dh}{dt} = \frac{h_\infty - h}{\tau_h}$$ B3.8

Parameter	Value	Definition
$\bar{g}_{Na}$	0.1152 nS pF^{-1}	Maximum conductance of I_{Na}

Table B3. Fast sodium current parameters

Voltage-dependent potassium current – I_{K1}

$$I_{K1} = \bar{g}_{K1} q (0.38 r_1 + 0.62 r_2)(V - E_K)$$ B4.1

$$E_K = \frac{RT}{F} ln\left(\frac{[K]_o}{[K]_i}\right)$$ B4.2

$$q_\infty = \frac{1}{1+e^{\frac{-V-7.7}{23.7}}}$$ B4.3

$$r_\infty = \frac{1}{1+e^{\frac{V+63}{6.3}}}$$ B4.4

$$\tau_q = \frac{500}{1+\left(\frac{V+60.71}{15.79}\right)^2}$$ B4.5

$$\tau_{r_1} = \frac{5\times10^3}{1+\left(\frac{V+62.71}{35.86}\right)^2}$$ B4.6

$$\tau_{r_2} = 3\times10^4 + \frac{2.2\times10^5}{1+e^{\left(\frac{V+22}{4}\right)}}$$ B4.7

$$\frac{dq}{dt} = \frac{q_\infty - q}{\tau_q}$$ B4.8

$$\frac{dr_1}{dt} = \frac{r_\infty - r_1}{\tau_{r_1}}$$ B4.9

$$\frac{dr_2}{dt} = \frac{r_\infty - r_2}{\tau_{r_2}}$$ B4.10

Parameter	Value	Definition
$\bar{g}_{K1}$	0.2048 nS pF^{-1}	Maximum conductance of I_{K1}

Table B4. Type-1 Voltage-dependent potassium current parameters

Voltage-dependent potassium current – I_{K2}

$$I_{K2} = \bar{g}_{K2}\,p(0.75k_1 + 0.25k_2)(V - E_K)$$ B5.1

$$E_K = \frac{RT}{F} ln\left(\frac{[K]_o}{[K]_i}\right)$$ B5.2

$$p_\infty = \frac{1}{1+e^{\frac{-V+4.2}{23}}}$$ B5.3

$$k_\infty = \frac{1}{1+e^{\frac{V+21.2}{5.7}}}$$ B5.4

$$\tau_p = \frac{100}{1+\left(\frac{V+64.1}{28.67}\right)^2}$$ B5.5

$$\tau_{k_1} = 1\times10^6 - \frac{1\times10^6}{\left(1+e^{\frac{V-315}{50}}\right)\left(1+e^{\frac{-V-74.9}{8}}\right)}$$ B5.6

$$\tau_{k_2} = 2.5\times10^6 - \frac{2.5\times10^6}{\left(1+e^{\frac{V-132.87}{25.4}}\right)\left(1+e^{\frac{-V-24.92}{2.68}}\right)}$$ B5.7

$$\frac{dp}{dt} = \frac{p_\infty - p}{\tau_p}$$ B5.8

$$\frac{dk_1}{dt} = \frac{k_\infty - k_1}{\tau_{k_1}}$$ B5.9

$$\frac{dk_2}{dt} = \frac{k_\infty - k_2}{\tau_{k_2}}$$ B5.10

Parameter	Value	Definition
$\bar{g}_{K2}$	0.0315 nS pF^{-1}	Maximum conductance of I_{K2}

Table B5. Type-2 Voltage-dependent potassium current parameters

A-type transient potassium current – I_{KA}

$$I_{KA} = \bar{g}_{KA} s x (V - E_K)$$ B6.1

$$E_K = \frac{RT}{F} ln\left(\frac{[K]_o}{[K]_\mathrm{i}}\right)$$ B6.2

$$s_\infty = \frac{1}{1+e^{\frac{-V-27.79}{7.57}}}$$ B6.3

$$x_\infty = 0.3293 + \frac{0.71165}{1+e^{\frac{V+53.919}{7.8111}}}$$ B6.4

$$\tau_s = \frac{17}{1+\left(\frac{V+20.52}{35}\right)^2}$$ B6.5

$$\tau_x = 7.5 + \frac{10}{1+\left(\frac{V+34.18}{120}\right)^2}$$ B6.6

$$\frac{ds}{dt} = \frac{s_\infty - s}{\tau_s}$$ B6.7

$$\frac{dx}{dt} = \frac{x_\infty - x}{\tau_x}$$ B6.8

Parameter	Value	Definition
$\bar{g}_{KA}$	0.001418 nS pF^{-1}	Maximum conductance of I_{KA}

Table B6. A-type transient potassium current parameters

Calcium-activated potassium current – I_{KCa}

$$I_{KCa} = \frac{A_c N_{BKCa} P_{KCA} i_{KCa}}{C_{est}}$$ B7.1

$$P_{KCa} = 0.17 p_f + 0.83 p_s$$ B7.2

$$i_{KCa} = 10^4 P_{BKCa} V \frac{F^2}{RT} \frac{[K]_\mathrm{o} - [K]_i\, e^{\frac{VF}{RT}}}{1-e^{\frac{VF}{RT}}}$$ B7.3

$$\bar{p}_o = \left(1 + e^{\frac{V_{1/2KCa} - V}{18.25}}\right)^{-1}$$ B7.4

$$V_{1/2KCa} = -41.7 log_{10}[Ca^{2+}]_i - 128.2$$ B7.5

$$\frac{dp_f}{dt} = \frac{\bar{p}_o - p_f}{\tau_{p_f}}$$ B7.6

$$\frac{dp_s}{dt} = \frac{\bar{p}_o - p_s}{\tau_{p_s}}$$ B7.7

Parameter	Value	Definition
N_{BKCa}	5.94 x 10^6 cm^{-2}	Channel density
C_{est}	100 pF	Estimated cell capacitance of late pregnant rat
τ_{p_f}	0.84 ms	Fast activation time constant
τ_{p_s}	35.9 ms	Slow activation time constant
P_{BKCa}	3.9 x 10^{-12} cm^3 s^{-1}	Single channel permeability

Table B7. Calcium-activated potassium current parameters

Background potassium leakage current – I_{Kleak}

$$I_{Kleak} = g_{K-leak}(V - E_K)$$ B8.1

Parameter	Value	Definition
g_{K-leak}	0.0004 nS pF^{-1}	Conductance of I_{Kleak}

Table B8. Background K^+ leakage current parameters

Hyperpolarisation-activated current – I_h

$$I_h = \bar{g}_h y(V - E_h)$$ B9.1

$$E_h = \frac{RT}{F} ln\left(\frac{[K]_o + (P_{Na}/P_K)[Na^+]_o}{[K]_i + (P_{Na}/P_K)[Na^+]_i}\right)$$ B9.2

$$y_\infty = \frac{1}{1+e^{\frac{V+105.39}{8.66}}}$$ B9.3

$$\tau_y = \frac{1}{3.5\times10^{-6}e^{-0.0497V} + 0.04e^{0.0521V}}$$ B9.4

$$\frac{dy}{dt} = \frac{y_\infty - y}{\tau_y}$$ B9.5

Parameter	Value	Definition
$\bar{g}_h$	0.04065 nS pF^{-1}	Maximum conductance of I_h
P_{Na}/P_K	0.35	Na^+ and K^+ permeability ratio

Table B9. Hyperpolarisation-activated current parameters

Calcium-activated chloride current – I_{Cl}

$$I_{Cl} = \begin{cases} g_{Cl} g_{fCa} \sqrt{c1}(V - E_{Cl}) & if\ V > 0 \\ g_{Cl} g_{fCa}(c2)n(V - E_{Cl}) & otherwise \end{cases}$$ B10.1

$$E_{Cl} = \begin{cases} -30 \; if \; V > 0 \\ -5 \; otherwise \end{cases}$$ B10.2

$$g_{Cl} = \begin{cases} \bar{g}_{Cl}(19.26 + 365.8e^{0.0564V}) \;\; if \; V \leq -20 \\ \bar{g}_{Cl}(0.752 + 0.0349e^{-0.414V}) \;\; otherwise \end{cases}$$ B10.3

$$g_{fCa} = \frac{[Ca^{2+}]_i}{[Ca^{2+}]_i + K_{d,Cl}}$$ B10.4

$$c_\infty = \frac{1}{1+e^{\frac{44.08-V}{13.7}}}$$ B10.5

$$n_\infty = \frac{1}{1+e^{\frac{V+56.97}{20.29}}}$$ B10.6

$$\tau_{c1} = 24 - \frac{16.88}{1+e^{\frac{48.14-V}{13}}}$$ B10.7

$$\tau_{c2} = 32 + \frac{81.54}{1+e^{\frac{43.51-V}{39.5}}}$$ B10.8

$$\tau_n = 0.69 \; ms$$ B10.9

$$\frac{dc1}{dt} = \frac{c_\infty - c1}{\tau_{c1}}$$ B10.10

$$\frac{dc2}{dt} = \frac{c_\infty - c2}{\tau_{c2}}$$ B10.11

$$\frac{dn}{dt} = \frac{n_\infty - n}{\tau_n}$$ B10.12

Parameter	Value	Definition
$\bar{g}_{Cl}$	0.1406 pA pF^{-1}	Maximum conductance of I_{Cl}
$K_{d,Cl}$	257 µM	Calcium required for half-activation in I_{Cl}

Table B10. Chloride current parameters

Calcium pump current - I_{Capump}

$$I_{Capump} = \frac{z_{Ca} F V_c}{C_m B_{Ca} A_c} \cdot \frac{J_{pCa}}{1+\left(\frac{K_{m,pCa}}{[Ca^{2+}]_i}\right)^{hpCa}}$$ B11.1

Parameter	Value	Definition
J_{pCa}	3.5 x 10^{-6} mM ms^{-1}	Maximum flux of I_{Capump}
$K_{m,pCa}$	0.5 µM	Half saturation of $[Ca^{2+}]_i$
$hpCa$	2	Hill coefficient

Table B11. Plasma membrane Ca^{2+}-ATPase current parameters

Sodium/potassium exchanger current – I_{NaK}

$$I_{NaK} = g_{NaK} f_{NaK} k_{NaK} n_{NaK}$$ B12.1

$$f_{NaK} = \frac{1}{1+0.125e^{\frac{-0.1VF}{RT}}+0.00219e^{\frac{[Na^+]_o}{49.71}}\cdot e^{\frac{-1.9VF}{RT}}}$$ B12.2

$$k_{NaK} = \frac{1}{1+\left(\frac{K_{d,K}}{[K^+]_o}\right)^{n_K}}$$ B12.3

$$n_{NaK} = \frac{1}{1+\left(\frac{K_{d,Na}}{[Na^+]_i}\right)^{n_{Na}}}$$ B12.4

Parameter	Value	Definition
g_{NaK}	3.4 nS pF^{-1}	Maximum conductance of I_{NaK}
$K_{d,K}$	2 mM	Half saturation of K^+ in I_{NaK}
n_K	1.5	Hill coefficient of K^+ in I_{NaK}
$K_{d,Na}$	22 mM	Half saturation of Na^+ in I_{NaK}
n_{Na}	2	Hill coefficient of Na^+ in I_{NaK}

Table B12. Sodium/potassium exchanger current parameters

Sodium/calcium exchanger current – I_{NaCa}

$$I_{NaCa} = \frac{J_{NaCa} z_{Ca} V_c F}{A_c C_m}$$ B13.1

$$J_{NaCa} = (Allo)(\Delta E)$$ B13.2

$$\Delta E = \frac{E_1}{E_2(E_3+E_4)}$$ B13.3

$$Allo = \frac{1}{1+\left(\frac{K_{m,Allo}}{[Ca^{2+}]_i}\right)^{n_{Allo}}}$$ B13.4

$$E_1 = \bar{J}_{NaCa}\left([Na^+]_i{}^3[Ca^{2+}]_0 e^{\frac{\gamma VF}{RT}} - [Na^+]_o{}^3[Ca^{2+}]_i e^{\frac{(\gamma-1)VF}{RT}}\right)$$ B13.5

$$E_2 = 1 + k_{sat} e^{\frac{(\gamma-1)VF}{RT}}$$ B13.6

$$E_3 = K_{m,Cao}[Na^+]_i{}^3 + K_{m,Nao}^3[Ca^{2+}]_i + K_{m,Nai}^3[Ca^{2+}]_o\left(1+\frac{[Ca^{2+}]_i}{K_{m,Cai}}\right)$$ B13.7

$$E_4 = [Ca^{2+}]_o[Na^+]_i{}^3 + [Na^+]_o{}^3[Ca^{2+}]_i + [Na^+]_o{}^3 K_{m,Cai}\left(1+\frac{[Na^+]_i{}^3}{K_{m,Nai}}\right)$$ B13.8

Parameter	Value	Definition
$\bar{J}_{NaCa}$	1.75 x 10^{-5} mM ms^{-1}	Maximum flux of Na^+ / Ca^{2+} exchanger
$K_{m,Allo}$	0.3 µM	Calcium activation of I_{NaCa}
n_{Allo}	4	Hill coefficient of I_{NaCa}
k_{sat}	0.27	Saturation of I_{NaCa} at negative potentials
γ	0.35	Electric field energy barrier of Na^+ / Ca^{2+} exchanger
$K_{m,Nai}$	30 mM	Saturation concentration of $[Na^+]_i$
$K_{m,Cai}$	7 µM	Saturation concentration of $[Ca^{2+}]_i$
$K_{m,Nao}$	87.5 mM	Saturation concentration of $[Na^+]_o$
$K_{m,Cao}$	1.3 mM	Saturation concentration of $[Ca^{2+}]_o$

Table B13. Sodium/calcium exchanger current parameters

Sodium-potassium-chloride co-transport current – I_{NaKCl}

$$I_{NaKCl} = -R_{NaKCl} z_{Cl} A_c L_{NaKCl} RFT \times ln\left(\frac{[Na^+]_o[K^+]_o}{[Na^+]_i[K^+]_i}\left(\frac{[Cl^-]_o}{[Cl^-]_i}\right)^2\right) \quad \text{B14.1}$$

$$I_{NaKCl-Na} = I_{NaKCl-K} = -0.5 I_{NaKCl-Cl} \quad \text{B14.2}$$

Parameter	Value	Definition
L_{NaKCl}	3.58 x 10^{-10} $nmol^{-2}$ J^{-1} s^{-1} cm^2	Cotransport coefficient
R_{NaKCl}	1	Cotransport regulation

Table B14. Sodium-potassium-chloride co-transport current parameters

Store-operated non-selective cation current – I_{SOC}

$$I_{SOC} = I_{SOCCa} + I_{SOCNa} \quad \text{B15.1}$$

$$I_{SOCNa} = g_{SOCNa} P_{SOC}(V - E_{Na}) \quad \text{B15.2}$$

$$I_{SOCCa} = g_{SOCCa} P_{SOC}(V - E_{Ca}) \quad \text{B15.3}$$

$$P_{SOC} = \left(1 + \frac{[Ca^{2+}]_i}{K_{m,SOC}}\right)^{-1} \quad \text{B15.4}$$

Parameter	Value	Definition
g_{SOCNa}	5.75 x 10^{-4} nS pF^{-1}	Maximum conductance of the Na^+ component of I_{SOC}
g_{SOCCa}	8.3 x 10^{-5} nS pF^{-1}	Maximum conductance of the Ca^{2+} component of I_{SOC}
$K_{m,SOC}$	0.1 µM	Half activation constant of I_{SOC}

Table B15. Store-operated non-selective cation current parameters

Non-selective cation current – I_{NSCC}

$$I_{NSCC} = I_{NSCa} + I_{NSNa} + I_{NSK} + I_{NSleak} \quad \text{B16.1}$$

$$I_{NSCa} = 0.15 f_{Mg} g_{[Ca^{2+}]_o} \bar{g}_{NS}(V - E_{NS})$$ B16.2

$$I_{NSNa} = f_{Mg} g_{[Na^{+}]_o} \bar{g}_{NS}(V - E_{NS})$$ B16.3

$$I_{NSK} = 1.19 f_{Mg} g_{[K^{+}]_o} \bar{g}_{NS}(V - E_{NS})$$ B16.4

$$I_{NSleak} = f_{Mg} \bar{g}_{leak}(V - E_{NS})$$ B16.5

$$g_{[X]_o} = \frac{1}{g_s} \frac{0.03}{1+\left(\frac{150}{[X]_o+10^{-8}}\right)^2}$$ B16.6

$$g_s = \begin{cases} 0.000525 \; for \; Ca^{2+} \\ 0.0123 \quad otherwise \end{cases}$$ B16.7

$$f_{Mg} = 0.1 + \frac{0.9}{1+\left(\frac{[Mg^{2+}]_o}{K_{d,Mg}}\right)^{1.3}}$$ B16.8

$$E_{NS} = \frac{RT}{F} \cdot ln\left(\frac{\frac{P_{Na}}{P_{Cs}}[Na^+]_o + \frac{P_K}{P_{Cs}}[K^+]_o + P'_{Ca}\frac{4P_{Ca}}{P_{Cs}}[Ca^{2+}]_o}{\frac{P_{Na}}{P_{Cs}}[Na^+]_i + \frac{P_K}{P_{Cs}}[K^+]_i + P'_{Ca}\frac{4P_{Ca}}{P_{Cs}}[Ca^{2+}]_i}\right)$$ B16.9

$$P'_{Ca} = \frac{P_{Ca}}{1+e^{\frac{VF}{RT}}}$$ B16.10

Parameter	Value	Definition
$\bar{g}_{NS}$	0.0123 nS pF^{-1}	Maximum conductance of I_{NSCC}
$\bar{g}_{leak}$	0.009685 nS pF^{-1}	Maximum conductance of leakage component of I_{NSCC}
$K_{d,Mg}$	0.28	Half saturation concentration
P_{Na}/P_{Cs}	0.9	Permeability ratio of Na^+ : Cs^+
P_K/P_{Cs}	1.3	Permeability ratio of K^+ : Cs^+
P_{Ca}/P_{Cs}	0.89	Permeability ratio of Ca^{2+} : Cs^+

Table B16. Store-operated non-selective cation current parameters

Ionic balance currents

$$I_{Ca-tot} = I_{CaL} + I_{CaT} + I_{NSCa} + I_{SOCCa} - 2I_{NaCa} + I_{Capump} + I_{up} - I_{rel}$$ B17.1

$$\frac{d[Ca^{2+}]_i}{dt} = -\frac{A_c C_m}{V_c z_{Ca} F}(I_{Ca-tot}) \times F_{BCa}\left(1 + \frac{[S_{CM}]K_d}{(K_d+[Ca^{2+}]_i)^2} + \frac{[B_F]K_{d,B}}{(K_{d,B}+[Ca^{2+}]_i)^2}\right)^{-1}$$ B17.2

$$I_{Na-tot} = I_{Na} + I_{NSNa} + I_{SOCNa} - 3I_{NaK} + 3I_{NaCa} + P_{Na}I_h - 0.5I_{NaKCl}$$ B17.3

$$\frac{d[Na^+]_i}{dt} = -\frac{A_c C_m}{V_c z_{Na} F}(I_{Na-tot})$$ B17.4

$$I_{K-tot} = I_{K1} + I_{K2} + I_{KA} + I_{KCa} - 2I_{NSK} + I_{Kleak} + I_{NaKCl} - 2I_{NaK}$$ B17.5

$$\frac{d[K^+]_i}{dt} = -\frac{A_c C_m}{V_c z_K F}(I_{K-tot})$$ B17.6

$$I_{Cl-tot} = I_{Cl} + I_{NaKCl}$$ B17.7

$$\frac{d[Cl^{+}]_i}{dt} = -\frac{A_c C_m}{V_c z_{Cl} F}(I_{Cl-tot})$$ B17.8

$$I_{tot} = I_h + I_{CaL} + I_{CaT} + I_{Na} + I_{K1} + I_{K2} + I_{KA} + I_{KCa} + I_{NSCC} + I_{Cl} + I_{Capump} + I_{NaK} + 0.5 I_{NaCa} + I_{sus} + I_{soc} + I_{Kleak} + I_{NaKCl}$$ B17.9

$$\frac{dV}{dt} = \frac{I_{tot}+I_{stim}}{C_m}$$ B17.10

Parameter	Value	Definition
C_m	1.4 μF cm^{-2}	Specific membrane capacitance
A_c	7.422 x 10^{-5} cm^2	Area of the cell
V_c	2.65 x 10^{-8} cm^3	Volume of the cell
z_{Ca}	2	Valency of Ca^{2+}
z_{Na}	1	Valency of Na^{+}
z_K	1	Valency of K^{+}
z_{Cl}	-1	Valency of Cl^{-}
F	96485 C M^{-1}	Faraday's constant
P_{Na}	0.35	Permeability of sodium ions
I_{sus}	0.6 pA	Sustained stimulus
I_{stim}	Various	Stimulus current
F_{BCa}	20	Buffer fractional strength factor
$[S_{CM}]$	0.3	Total concentration of calmodulin sites for Ca^{2+}
$[B_F]$	0.2	Total concentration of other buffer sites for Ca^{2+}
K_d	2.6 x 10^{-4}	Dissociation constant
$K_{d,B}$	5.3981 x 10^{-4}	Dissociation constant in buffer

Table B17. Ionic balance parameters

Sarcoplasmic reticulum currents – I_{up}, I_{tr}, I_{rel}

$$I_{up} = \bar{I}_{up}\frac{[Ca^{2+}]_i}{([Ca^{2+}]_i + K_{m,up})}$$ B18.1

$$I_{tr} = \frac{([Ca^{2+}]_u - [Ca^{2+}]_r) z_{Ca} F vol_u}{\tau_{tr}}$$ B18.2

$$I_{rel} = F_{rel}\frac{(R_{10}{}^2 + R_{leak})([Ca^{2+}]_r - [Ca^{2+}]_i) z_{Ca} F vol_r}{\tau_{rel}}$$ B18.3

$$\frac{d[Ca^{2+}]_u}{dt} = \frac{I_{up} - I_{tr}}{z_{Ca} F vol_u}$$ B18.4

$$\frac{d[Ca^{2+}]_r}{dt} = \frac{I_{tr} - I_{rel}}{z_{Ca} F vol_r}\left(1 + \frac{\overline{[CSQN]} K_{CSQN}}{(K_{CSQN} + [Ca^{2+}]_r)^2}\right)^{-1}$$ B18.5

Parameter	Value	Definition
$\bar{I}_{up}$	0.668 pA	Max SR uptake current
$K_{m,up}$	10 µM	Michaelis constant of SERCA pump
vol_u	1.33 x 10^{-9} cm^3	Volume of uptake compartment
vol_r	1.33 x 10^{-11} cm^3	Volume of release compartment
τ_{tr}	180 ms	Time constant of I_{tr}
τ_{rel}	0.015 ms	Time constant of I_{rel}
$\overline{[CSQN]}$	15 mM	Concentration of calsequestrin in release compartment
K_{CSQN}	0.8 mM	Binding affinity of calsequestrin
R_{leak}	75.07 x 10^{-4}	Release compartment calcium leak rate

Table B18. SR parameters

Ryanodine receptors

$$\frac{dR_{00}}{dt} = -(K_{r1}{Ca_i}^2 + K_{r2}Ca_i)R_{00} \quad \text{B19.1}$$

$$\frac{dR_{10}}{dt} = K_{r1}{Ca_i}^2R_{00} - (K_{-r1} + K_{r2}Ca_i)R_{10} + K_{-r2}R_{11} \quad \text{B19.2}$$

$$\frac{dR_{01}}{dt} = K_{r2}Ca_iR_{00} + K_{-r1}R_{11} - (K_{-r2} + K_{r1}{Ca_i}^2)R_{01} \quad \text{B19.3}$$

$$\frac{dR_{11}}{dt} = K_{r2}Ca_iR_{10} - (K_{-r1} + K_{-r2})R_{11} + K_{r1}{Ca_i}^2R_{01} \quad \text{B19.4}$$

Parameter	Value	Definition
K_{r1}	2500 mM^{-2} ms^{-1}	Binding activation rate constant
K_{-r1}	0.0076 mM^{-1} ms^{-1}	Unbinding activation rate constant
K_{r2}	1.05 ms^{-1}	Binding inactivation rate constant
K_{-r2}	0.084 ms^{-1}	Unbinding inactivation rate constant

Table B19. RyR parameters

Kinetic cross-bridge cycling model

$$\frac{dM}{dt} = -K_1M + K_2Mp + K_7AM \quad \text{B20.1}$$

$$\frac{dMp}{dt} = K_4AMp + K_1M - (K_2 + K_3)Mp \quad \text{B20.2}$$

$$\frac{dAMp}{dt} = K_3Mp + K_6AM - (K_4 + K_5)AMp \quad \text{B20.3}$$

$$\frac{dAM}{dt} = K_5AMp - (K_7 + K_6)AM \quad \text{B20.4}$$

$$MLCK = \frac{MLCK_{max}}{1+\left(\frac{[Ca^{2+}]_i}{K_{MLCK}}\right)^{pM}} \quad \text{B20.5}$$

$$K_1 = C_{ms}\frac{MLCK}{1+\left(\frac{K_{Ca,MLCK}}{[Ca^{2+}]_i}\right)^{nM}} \quad \text{B20.6}$$

$K_6 = K_1$ B20.7

$Phosporylation\ fraction = AMp + Mp$ B20.8

$Stress\ fraction = AMp + AM$ B20.9

Parameter	Value	Definition
$MLCK_{max}$	0.84	Maximum fraction of MLCK
K_{MLCK}	721.746 nM	Half-activation of MLCK
$K_{Ca,MLCK}$	1.080 µM	Half-activation of MLCK set by $[Ca^{2+}]_i$
p_M	1	Hill coefficient for MLCK
n_M	8.7613	Hill coefficient for K_1
K_2	0. 4 ms^{-1}	Myosin dephosphorylation rate constant
K_3	1.8 ms^{-1}	Cross-bridge formation rate constant
K_4	0.1 ms^{-1}	Cross-bridge detachment rate constant
K_5	0.4 ms^{-1}	Attached myosin dephosphorylation rate constant
K_7	0.045 ms^{-1}	Latch state detachment rate constant

Table B20. Kinetic cross-bridge cycling parameters

Mechanical production model

$$\frac{dl_s}{dt} = \frac{1}{\mu_s}\left(F_s - k_s e^{\frac{\alpha_s(l_s - l_{s0})}{l_{s0}}} - 1\right)$$ B21.1

$$\frac{dl_a}{dt} = \frac{F_a e^{\beta\frac{(l_a - l_{opt})^2}{l_{opt}}} - f_{AMp}AMpv_x}{f_{AM}AM + f_{AMp}AMp}$$ B21.2

$$\frac{dl_x}{dt} = \frac{(k_{x1}AMp + k_{x2}AM)(l_c - l_a - l_s) - (f_{AMp}AMpv_x)}{f_{AM}AM + f_{AMp}AMp}$$ B21.3

$$l_x = l_c - l_a - l_s$$ B21.4

$$F_p = k_p\left(e^{\frac{\alpha_p(l_c - l_{c0})}{l_{c0}}} - 1\right)$$ B21.5

$$F_x = (k_{x1}AMp + k_{x2}AM)l_x e^{-\beta\left(\frac{l_a - l_{opt}}{l_{opt}}\right)^2}$$ B21.6

$$F_a = \left(f_{AMp}AMp\left(v_x + \frac{dl_a}{dt}\right) + f_{AM}AM\frac{dl_a}{dt}\right)e^{-\beta\left(\frac{l_a - l_{opt}}{l_{opt}}\right)^2}$$ B21.7

$$F_s = F_a$$ B21.8

$$F_t = F_a + F_p$$ B21.9

Parameter	Value	Definition
l_c	120 µm	Length of whole cell
l_{c0}	40 µm	Length of cell at zero passive force
l_{s0}	30 µm	Length of series viscoelastic component at zero force
l_{opt}	100 µm	Optimal length of active contractile component
k_{x1}	12.5 µN µm^{-1}	Phosphorylated cross-bridge stiffness constant
k_{x2}	8.8 µN µm^{-1}	Latch bridge stiffness constant
k_s	0.2 µN	Series element stiffness constant
k_p	0.1 µN	Parallel element stiffness constant
v_x	5 µm ms^{-1}	Cross-bridge cycling velocity
f_{AMp}	1.3 µN ms µm^{-1}	Friction constant for phosphorylated cross-bridges
f_{AM}	85.5 µN ms µm^{-1}	Friction constant for latch bridges
μ_s	0.01 µN ms µm^{-1}	Viscosity coefficient of series element
α_s	4.5	Length modulation for series viscoelastic element
α_p	0.1	Length modulation for passive element
β	7.5	Length modulation constant for active and cross-bridge elements

Table B21. Force production parameters

Parameter	Value	Definition
T	308 K	Model temperature
F	96485 mC mmol^{-1}	Faraday constant
R	8314 µJ K^{-1} mmol^{-1}	Molar gas constant
z_{Na}	1	Valency of Na^+
z_K	1	Valency of K^+
z_{Ca}	2	Valency of Ca^{2+}
z_{Cl}	-1	Valency of Cl^-
$[Na^+]_o$	140 mM	Na^+ extracellular concentration
$[K^+]_o$	5.4 mM	K^+ extracellular concentration
$[Ca^{2+}]_o$	2.5 mM	Ca^{2+} extracellular concentration
$[Mg^{2+}]_o$	0.5 mM	Mg^{2+} extracellular concentration
$[Cl^-]_o$	146.4 mM	Cl^- extracellular concentration
C_m	1.4 µF cm^{-2}	Specific membrane capacitance
A_c	7.422 x 10^{-5} cm^2	Area of the cell
V_c	2.65 x 10^{-8} cm^3	Volume of the cell

Table B22. Other parameters

Supplement C – Initial Conditions

This supplement contains the initial conditions used in our single cell model.

Parameter	Value	Definition
$[Ca^{2+}]_i$	9.43 x 10^{-5} mM	Intracellular calcium concentration
$[Ca^{2+}]_o$	2.5 mM	Extracellular calcium concentration
$[Na^{+}]_i$	4 mM	Intracellular sodium concentration
$[Na^{+}]_o$	140 mM	Extracellular sodium concentration
$[K^{+}]_i$	140 mM	Intracellular potassium concentration
$[K^{+}]_o$	5.4 mM	Extracellular potassium concentration
$[Cl^{-}]_i$	130 mM	Intracellular chloride concentration
$[Cl^{-}]_o$	146.6 mM	Extracellular chloride concentration
$[Mg^{2+}]_o$	0.5 mM	Extracellular magnesium concentration

Table C1. Ionic concentrations

Parameter	Value	Definition
d	0.0133195	I_{CaL} activation gate
$f1$	0.938986	I_{CaL} inactivation gate, fast
$f2$	0.938986	I_{CaL} inactivation gate, slow
b	0.179073	I_{CaT} activation gate
g	0.0308496	I_{CaT} inactivation gate
m	0.129659	I_{Na} activation gate
h	0.360711	I_{Na} inactivation gate
q	0.0741461	I_{K1} activation gate
$r1$	0.151067	I_{K1} inactivation gate, fast
$r2$	0.108987	I_{K1} inactivation gate, slow
p	0.0794842	I_{K2} activation gate
$k1$	0.995353	I_{K2} inactivation gate, fast
$k2$	0.954862	I_{K2} inactivation gate, slow
s	0.0385646	I_{KA} activation gate
x	0.644685	I_{KA} inactivation gate
y	0.00212991	I_{h} activation gate
$c1$	0.00089031	I_{Cl} activation gate, fast
$c2$	0.00089031	I_{Cl} activation gate, slow
n	0.440717	I_{Cl} inactivation gate

Table C2. Gating variables

Parameter	Value	Definition
l_s	30.08 µm	Length of spring component of cell
l_a	25.3 µm	Length of active component of cell
M	0.97	Detached dephosphorylated cross-bridge fraction
Mp	0.01	Detached phosphorylated cross-bridge fraction
AMp	0.01	Attached phosphorylated cross-bridge fraction
AM	0.01	Attached dephosphorylated cross-bridge fraction

Table C3. Kinetics

References

[1] H. C. Parkington, M. A. Tonta, S. P. Brennecke, and H. A. Coleman, “Contractile activity, membrane potential, and cytoplasmic calcium in human uterine smooth muscle in the third trimester of pregnancy and during labor,” *Am. J. Obstet. Gynecol.*, vol. 181, no. 6, pp. 1445–1451, Dec. 1999.

[2] H. Miyoshi, T. Urabe, and A. Fujiwara, “Electrophysiological properties of membrane currents in single myometrial cells isolated from pregnant rats,” *Pflüg. Arch. Eur. J. Physiol.*, vol. 419, no. 3–4, pp. 386–393, Oct. 1991.

[3] N. Sperelakis, Y. Inoue, and Y. Ohya, “Fast Na+ channels and slow Ca2+ current in smooth muscle from pregnant rat uterus,” *Mol. Cell. Biochem.*, vol. 114, no. 1–2, pp. 79–89, Sep. 1992.

[4] R. C. Young, L. H. Smith, and M. D. McLaren, “T-type and L-type calcium currents in freshly dispersed human uterine smooth muscle cells,” *Am. J. Obstet. Gynecol.*, vol. 169, no. 4, pp. 785–792, Oct. 1993.

[5] A. V. Shmigol, D. A. Eisner, and S. Wray, “Properties of Voltage-Activated [Ca2+]i Transients in Single Smooth Muscle Cells Isolated from Pregnant Rat Uterus,” *J. Physiol.*, vol. 511, no. 3, pp. 803–811, Sep. 1998.

[6] T. Yamamoto, “Effects of estrogens on Ca channels in myometrial cells isolated from pregnant rats,” *Am. J. Physiol.*, vol. 268, no. 1 Pt 1, pp. C64-69, Jan. 1995.

[7] T. F. McDonald, S. Pelzer, W. Trautwein, and D. J. Pelzer, “Regulation and modulation of calcium channels in cardiac, skeletal, and smooth muscle cells,” *Physiol. Rev.*, vol. 74, no. 2, pp. 365–507, Apr. 1994.

[8] M. Yoshino, S. Y. Wang, and C. Y. Kao, “Sodium and Calcium Inward Currents in Freshly Dissociated Smooth Myocytes of Rat Uterus,” *J. Gen. Physiol.*, vol. 110, no. 5, pp. 565–577, Nov. 1997.

[9] K. Jones, A. Shmygol, S. Kupittayanant, and S. Wray, “Electrophysiological characterization and functional importance of calcium-activated chloride channel in rat uterine myocytes,” *Pflüg. Arch. Eur. J. Physiol.*, vol. 448, no. 1, pp. 36–43, Apr. 2004.

[10] A. M. Blanks, Z.-H. Zhao, A. Shmygol, G. Bru-Mercier, S. Astle, and S. Thornton, “Characterization of the molecular and electrophysiological properties of the T-type calcium channel in human myometrium,” *J. Physiol.*, vol. 581, no. Pt 3, pp. 915–926, Jun. 2007.

[11] J. R. Serrano, E. Perez-Reyes, and S. W. Jones, “State-dependent inactivation of the alpha1G T-type calcium channel,” *J. Gen. Physiol.*, vol. 114, no. 2, pp. 185–201, Aug. 1999.

[12] J. Hering, A. Feltz, and R. C. Lambert, “Slow Inactivation of the CaV3.1 Isotype of T-Type Calcium Channels,” *J. Physiol.*, vol. 555, no. 2, pp. 331–344, Mar. 2004.

[13] K. T. Asokan, S. N. Sarkar, S. K. Mishra, and V. Raviprakash, “Effects of mibefradil on uterine contractility,” *Eur. J. Pharmacol.*, vol. 455, no. 1, pp. 65–71, Nov. 2002.

[14] S.-E. Lee, D.-S. Ahn, and Y.-H. Lee, “Role of T-type Ca Channels in the Spontaneous Phasic Contraction of Pregnant Rat Uterine Smooth Muscle,” *Korean J. Physiol. Pharmacol. Off. J. Korean Physiol. Soc. Korean Soc. Pharmacol.*, vol. 13, no. 3, pp. 241–249, Jun. 2009.

[15] T. Ohkubo, T. Kawarabayashi, Y. Inoue, and K. Kitamura, “Differential expression of L- and T-type calcium channels between longitudinal and circular muscles of the rat myometrium during pregnancy,” *Gynecol. Obstet. Invest.*, vol. 59, no. 2, pp. 80–85, 2005.

[16] A. K. Grover, C. Y. Kwan, and E. E. Daniel, “Na--Ca exchange in rat myometrium membrane vesicles highly enriched in plasma membranes,” *Am. J. Physiol.*, vol. 240, no. 5, pp. C175-182, May 1981.

[17] A. K. Grover, C. Y. Kwan, P. K. Rangachari, and E. E. Daniel, “Na-Ca exchange in a smooth muscle plasma membrane-enriched fraction,” *Am. J. Physiol.*, vol. 244, no. 3, pp. C158-165, Mar. 1983.

[18] A. K. Grover and C. Y. Kwan, “High affinity Ca-binding to smooth muscle plasma membrane: inhibition by cations,” *Cell Calcium*, vol. 7, no. 3, pp. 187–192, Jun. 1986.

[19] “Computational Cell Biology.” [Online]. Available: http://www.springer.com/new+%26+forthcoming+titles+(default)/book/978-0-387-95369-4. [Accessed: 04-Jul-2013].

[20] G. S. Taylor, D. M. Paton, and E. E. Daniel, “Characteristics of electrogenic sodium pumping in rat myometrium,” *J. Gen. Physiol.*, vol. 56, no. 3, pp. 360–375, Sep. 1970.

[21] A. Turi, Z. Marcsek, N. Müllner, M. Kucsera, and Z. Bori, “The activity of Na+/K(+)-ATPase and abundance of its mRNA are regulated in rat myometrium during pregnancy,” *Biochem. Biophys. Res. Commun.*, vol. 188, no. 3, pp. 1191–1197, Nov. 1992.

[22] C. H. Luo and Y. Rudy, “A dynamic model of the cardiac ventricular action potential. II. Afterdepolarizations, triggered activity, and potentiation,” *Circ. Res.*, vol. 74, no. 6, pp. 1097–1113, Jun. 1994.

[23] T. Amédée, J. F. Renaud, K. Jmari, A. Lombet, J. Mironneau, and M. Lazdunski, “The presence of Na+ channels in myometrial smooth muscle cells is revealed by specific neurotoxins,” *Biochem. Biophys. Res. Commun.*, vol. 137, no. 2, pp. 675–681, Jun. 1986.
[24] I. A. Greenwood and N. Leblanc, “Overlapping pharmacology of Ca2+-activated Cl- and K+ channels,” *Trends Pharmacol. Sci.*, vol. 28, no. 1, pp. 1–5, Jan. 2007.
[25] Y. Inoue and N. Sperelakis, “Gestational change in Na+ and Ca2+ channel current densities in rat myometrial smooth muscle cells,” *Am. J. Physiol.*, vol. 260, no. 3 Pt 1, pp. C658-663, Mar. 1991.
[26] Y. Abe, “Effects of changing the ionic environment on passive and active membrane properties of pregnant rat uterus,” *J. Physiol.*, vol. 214, no. 1, pp. 173–190, Apr. 1971.
[27] T. J. Hund and Y. Rudy, “Rate Dependence and Regulation of Action Potential and Calcium Transient in a Canine Cardiac Ventricular Cell Model,” *Circulation*, vol. 110, no. 20, pp. 3168–3174, Nov. 2004.
[28] G. A. Knock, S. V. Smirnov, and P. I. Aaronson, “Voltage-gated K+ currents in freshly isolated myocytes of the pregnant human myometrium,” *J. Physiol.*, vol. 518, no. Pt 3, pp. 769–781, Aug. 1999.
[29] S. Y. Wang, M. Yoshino, J. L. Sui, M. Wakui, P. N. Kao, and C. Y. Kao, “Potassium currents in freshly dissociated uterine myocytes from nonpregnant and late-pregnant rats,” *J. Gen. Physiol.*, vol. 112, no. 6, pp. 737–756, Dec. 1998.
[30] G. A. Knock, R. M. Tribe, A. A. Hassoni, and P. I. Aaronson, “Modulation of potassium current characteristics in human myometrial smooth muscle by 17beta-estradiol and progesterone,” *Biol. Reprod.*, vol. 64, no. 5, pp. 1526–1534, May 2001.
[31] A. Kapela, A. Bezerianos, and N. M. Tsoukias, “A mathematical model of Ca2+ dynamics in rat mesenteric smooth muscle cell: agonist and NO stimulation,” *J. Theor. Biol.*, vol. 253, no. 2, pp. 238–260, Jul. 2008.
[32] K. Okabe, Y. Inoue, T. Kawarabayashi, H. Kajiya, F. Okamoto, and H. Soeda, “Physiological significance of hyperpolarization-activated inward currents (Ih) in smooth muscle cells from the circular layers of pregnant rat myometrium,” *Pflüg. Arch. Eur. J. Physiol.*, vol. 439, no. 1–2, pp. 76–85, Dec. 1999.
[33] H. Satoh, “Identification of a hyperpolarization-activated inward current in uterine smooth muscle cells during pregnancy,” *Gen. Pharmacol.*, vol. 26, no. 6, pp. 1335–1338, Oct. 1995.
[34] M. H. Zhu, T. S. Sung, K. O’Driscoll, S. D. Koh, and K. M. Sanders, “Intracellular Ca(2+) release from endoplasmic reticulum regulates slow wave currents and pacemaker activity of interstitial cells of Cajal,” *Am. J. Physiol. Cell Physiol.*, vol. 308, no. 8, pp. C608-620, Apr. 2015.
[35] R. Bao, L. M. Lifshitz, R. A. Tuft, K. Bellvé, K. E. Fogarty, and R. ZhuGe, “A Close Association of RyRs with Highly Dense Clusters of Ca2+-activated Cl− Channels Underlies the Activation of STICs by Ca2+ Sparks in Mouse Airway Smooth Muscle,” *J. Gen. Physiol.*, vol. 132, no. 1, pp. 145–160, Jul. 2008.
[36] K. Bernstein *et al.*, “Calcium-activated chloride channels anoctamin 1 and 2 promote murine uterine smooth muscle contractility,” *Am. J. Obstet. Gynecol.*, vol. 211, no. 6, p. 688.e1-10, Dec. 2014.
[37] R. C. Young and A. Bemis, “Calcium-activated chloride currents prolongs the duration of contractions in pregnant rat myometrial tissue,” *Reprod. Sci. Thousand Oaks Calif*, vol. 16, no. 8, pp. 734–739, Aug. 2009.
[38] J. Berg, H. Yang, and L. Y. Jan, “Ca2+-activated Cl− channels at a glance,” *J. Cell Sci.*, vol. 125, no. 6, pp. 1367–1371, Mar. 2012.
[39] A. Mijušković, A. N. Kokić, Z. O. Dušić, M. Slavić, M. B. Spasić, and D. Blagojević, “Chloride channels mediate sodium sulphide-induced relaxation in rat uteri,” *Br. J. Pharmacol.*, vol. 172, no. 14, pp. 3671–3686, Jul. 2015.
[40] I. Varga, L. Urban, M. Kajanová, and Š. Polák, “Functional histology and possible clinical significance of recently discovered telocytes inside the female reproductive system,” *Arch. Gynecol. Obstet.*, May 2016.
[41] R. C. Young, “Myocytes, myometrium, and uterine contractions,” *Ann. N. Y. Acad. Sci.*, vol. 1101, pp. 72–84, Apr. 2007.
[42] G. M. Billig, B. Pál, P. Fidzinski, and T. J. Jentsch, “Ca2+-activated Cl- currents are dispensable for olfaction,” *Nat. Neurosci.*, vol. 14, no. 6, pp. 763–769, Jun. 2011.
[43] K. Wang, C. Chen, J. Ma, J. Lao, and Y. Pang, “Contribution of calcium-activated chloride channel to elevated pulmonary artery pressure in pulmonary arterial hypertension induced by high pulmonary blood flow,” *Int. J. Clin. Exp. Pathol.*, vol. 8, no. 1, pp. 146–154, 2015.
[44] N. Leblanc *et al.*, “Molecular and functional significance of Ca2+-activated Cl− channels in pulmonary arterial smooth muscle,” *Pulm. Circ.*, vol. 5, no. 2, pp. 244–268, Jun. 2015.
[45] B. M. Sanborn, “Relationship of ion channel activity to control of myometrial calcium,” *J. Soc. Gynecol. Investig.*, vol. 7, no. 1, pp. 4–11, Feb. 2000.

[46] M. J. Taggart and S. Wray, “Contribution of sarcoplasmic reticular calcium to smooth muscle contractile activation: gestational dependence in isolated rat uterus,” *J. Physiol.*, vol. 511, no. Pt 1, pp. 133–144, Aug. 1998.

[47] S. Kupittayanant, M. j. m. Luckas, and S. Wray, “Effect of inhibiting the sarcoplasmic reticulum on spontaneous and oxytocin-induced contractions of human myometrium,” *BJOG Int. J. Obstet. Gynaecol.*, vol. 109, no. 3, pp. 289–296, Mar. 2002.

[48] K. Noble and S. Wray, “The role of the sarcoplasmic reticulum in neonatal uterine smooth muscle: enhanced role compared to adult rat,” *J. Physiol.*, vol. 545, no. Pt 2, pp. 557–566, Dec. 2002.

[49] K. Noble, A. Matthew, T. Burdyga, and S. Wray, “A review of recent insights into the role of the sarcoplasmic reticulum and Ca entry in uterine smooth muscle,” *Eur. J. Obstet. Gynecol. Reprod. Biol.*, vol. 144 Suppl 1, pp. S11-19, May 2009.

[50] D. Noble, L. Borysova, S. Wray, and T. Burdyga, “Store-operated Ca^{2+} entry and depolarization explain the anomalous behaviour of myometrial SR: effects of SERCA inhibition on electrical activity, Ca^{2+} and force,” *Cell Calcium*, vol. 56, no. 3, pp. 188–194, Sep. 2014.

[51] F. C. Loftus, M. J. E. Richardson, and A. Shmygol, “Fine spatiotemporal calcium signals and kinematic properties revealed by motion-corrected calcium images of contracting myometrium,” University of Warwick, Coventry, 2015.

[52] J. Yang, J. W. Clark, R. M. Bryan, and C. Robertson, “The myogenic response in isolated rat cerebrovascular arteries: smooth muscle cell model,” *Med. Eng. Phys.*, vol. 25, no. 8, pp. 691–709, Oct. 2003.

[53] C. M. Hai and R. A. Murphy, “Cross-bridge phosphorylation and regulation of latch state in smooth muscle,” *Am. J. Physiol.*, vol. 254, no. 1 Pt 1, pp. C99-106, Jan. 1988.

[54] C. Y. Choi, “A biophysically detailed mathematical model of a single late pregnant rat myometrial cell (Manchester eScholar - The University of Manchester).” [Online]. Available: https://www.escholar.manchester.ac.uk/uk-ac-man-scw:110633. [Accessed: 08-Mar-2012].

[55] S. Wray, U. Ravens, A. Verkhratsky, and D. Eisner, “Two centuries of excitation-contraction coupling.,” *Cell Calcium*, vol. 35, no. 6, pp. 485–489, 2004.

[56] S. Wray, “Insights into the uterus,” *Exp. Physiol.*, vol. 92, no. 4, pp. 621–631, Jul. 2007.

[57] P. Mollard, J. Mironneau, T. Amedee, and C. Mironneau, “Electrophysiological characterization of single pregnant rat myometrial cells in short-term primary culture,” *Am. J. Physiol.*, vol. 250, no. 1 Pt 1, pp. C47-54, Jan. 1986.

[58] W. R. Loewenstein, “Junctional intercellular communication: the cell-to-cell membrane channel,” *Physiol. Rev.*, vol. 61, no. 4, pp. 829–913, Oct. 1981.
